# Influence of Headgroups in Ethylene-Tetrafluoroethylene-Based Radiation-Grafted Anion Exchange Membranes for CO_2_ Electrolysis

**DOI:** 10.1021/acssuschemeng.2c06205

**Published:** 2023-01-18

**Authors:** Carlos A. Giron Rodriguez, Bjørt Óladottir Joensen, Asger Barkholt Moss, Gastón O. Larrazábal, Daniel K. Whelligan, Brian Seger, John R. Varcoe, Terry R. Willson

**Affiliations:** † Surface Physics and Catalysis (SurfCat) Section, Department of Physics, Technical University of Denmark, 2800 Kgs. Lyngby, Denmark; ‡ School of Chemistry and Chemical Engineering, University of Surrey, Guildford GU2 7XH, U.K.

**Keywords:** electrochemical CO_2_ reduction, anion exchange membrane (AEM), cationic functional group, ion transport, zero-gap approach, ion exchange capacity

## Abstract

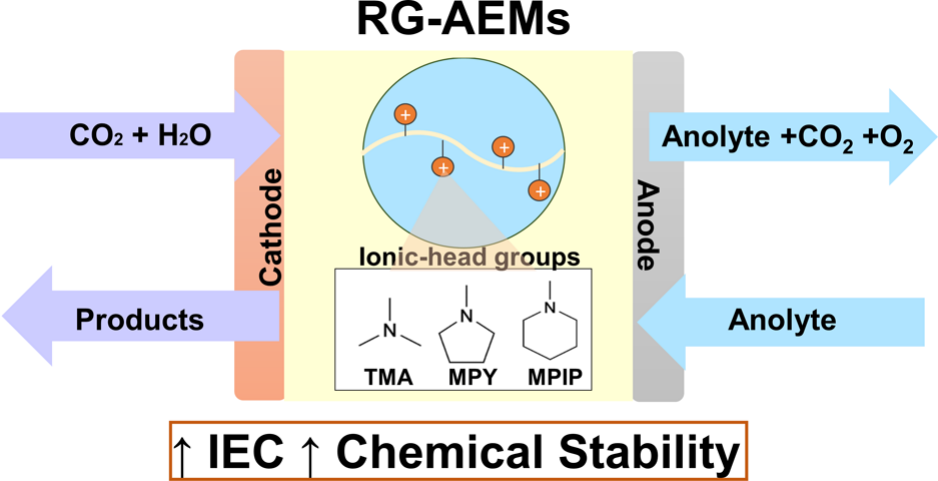

The performance of zero-gap CO_2_ electrolysis (CO_2_E) is significantly influenced by the membrane’s chemical structure and physical properties due to its effects on the local reaction environment and water/ion transport. Radiation-grafted anion-exchange membranes (RG-AEM) have demonstrated high ionic conductivity and durability, making them a promising alternative for CO_2_E. These membranes were fabricated using two different thicknesses of ethylene-tetrafluoroethylene polymer substrates (25 and 50 μm) and three different headgroup chemistries: benzyl-trimethylammonium, benzyl-*N*-methylpyrrolidinium, and benzyl-*N*-methylpiperidinium (MPIP). Our membrane characterization and testing in zero-gap cells over Ag electrocatalysts under commercially relevant conditions showed correlations between the water uptake, ionic conductivity, hydration, and cationic-head groups with the CO_2_E efficiency. The thinner 25 μm-based AEM with the MPIP-headgroup (ion-exchange capacities of 2.1 ± 0.1 mmol g^–1^) provided balanced in situ test characteristics with lower cell potentials, high CO selectivity, reduced liquid product crossover, and enhanced water management while maintaining stable operation compared to the commercial AEMs. The CO_2_ electrolyzer with an MPIP-AEM operated for over 200 h at 150 mA cm^–2^ with CO selectivities up to 80% and low cell potentials (around 3.1 V) while also demonstrating high conductivities and chemical stability during performance at elevated temperatures (above 60 °C).

## Abstract 2

The influence of the ionic head group on the radiation-grafted anion-exchange membrane properties is correlated to CO_2_E performance.

## Introduction

Driven by renewable sources, CO_2_ electrolysis (CO_2_E) is a promising approach to convert greenhouse gases into chemical feedstocks, providing a decentralized alternative to help close the carbon cycle.^1−4^ However, intensifying the operation at an industrial scale requires high current densities (>100 mA cm^–2^) and a long-term stable system to ensure low capital costs and techno-economic viability.^5−7^ The use of gas-diffusion electrodes (GDEs) in membrane electrode assemblies (MEAs) or flow-cell configurations has proven to perform effectively under commercially relevant conditions, overcoming the CO_2_ transport limitations inherent to fundamental H-cell research studies.^5,8−10^

While recent studies have focused on improving catalytic activity and selectivity of CO_2_ reduction,^11−14^ durability, and anode activity,^15−17^ lesser attention has been given to the membrane, yet this contributes substantially to the overall cell voltage and electrolyzer performance.^18−20^ Understanding how the ionomeric components affect water/ion transport mechanisms will enable improvements in stable CO_2_E operation, providing new tools for future research and development.

Ion exchange membranes (IEMs) typically have charged functional groups branching off a polymeric backbone.^21^ Anion exchange membranes (AEMs) have positively charged functional groups, allowing negatively charged ions to conduct, whereas cation exchange membranes (CEMs) have negatively charged functional groups, allowing cations to conduct. In terms of CO_2_E, if the catalyst is not in direct contact with the ionomeric components (e.g., flow cells with a catholyte layer), they do not affect catalytic selectivity.^22^ However, having an electrolyte between the catalyst and membrane will add a substantial overpotential, and thus, it is more efficient to have an intimate catalyst/membrane interaction (e.g., an MEA configuration). CEMs are well known to promote mainly hydrogen evolution reaction (HER), whereas AEMs allow high selectivity to CO_2_ reduction products. Conversely, AEMs have the issue where they primarily conduct CO_2_-derived HCO_3_^–^/CO_3_^2–^ anions (rather than OH^–^ anions) during CO_2_ electrolysis, leading to an anodic product gas comprising a mixture of CO_2_ and O_2_, effectively mitigating the environmental benefit from developing CO_2_E technologies.^23^ It is generally believed that solving this problem is more straightforward than improving the poor selectivities obtained with CEMs; thus, AEM-based cells currently appear most promising for CO_2_ electrolysis. The state-of-the-art CO_2_E cells with AEMs have reached high selectivities toward carbon-derived products (>95% for CO and 80% for C_2+_ products)^23^ and low cell potentials (<3.0 V) at high current densities (>100 mA cm^–2^), but they exhibit limited stability.^24^

The AEMs in this study were fabricated using another approach known as radiation grafting. This method involves the exposure of commercial substrate films to high-energy radiation to generate active sites (peroxide groups) that co-polymerize functional vinyl monomers to produce radiation-grafted copolymers (RG).^25^ The advantages of the radiation-grafting method include the preparation of IEMs without the need for a film formation step, the ability to compare different headgroup chemistries by post-graft functionalization of a single batch of RG membranes (with a single degree of grafting), yielding RG-IEMs with comparable ion-exchange capacities (IECs),^26^ and the ability to modify substrate films of the same type/chemistry (e.g., polyethylene) but with different intrinsic morphologies (crystallinities), resulting in IEMs with tailored water uptakes (WU) and transport properties.^27^

The mechanical and electrochemical properties of AEMs are known to be influenced by the cationic headgroups and the nature of the polymeric backbone.^28−30^ The development of hydrated ionic domains in IEMs governs the ionic and water transport during CO_2_E^31−33^ and will affect water management in the system. Optimizing such attributes will prevent electrode flooding, maintain adequate conductivity, and minimize the levels of salt precipitation at the cathode.^34,35^

Parameters like the WU and the hydration number (λ, the number of water molecules per ion-exchange site) are linked to water transport and can influence product crossover and ion mobility.^20,36^ The IEC (defined as the amount of charged groups bound to the polymer normalized to the mass of the dehydrated membrane in a specific anion form) is a fundamental characterization metric for IEM performance, which can often be correlated with ionic conductivity or water transport properties. Even though higher IECs are often desired, excessive amounts of cationic groups can disproportionally increase the WU, causing excessive swelling, which will dilute the charge carrier concentration and reduce conductivity and mechanical robustness.^37^ In general, AEMs for CO_2_ electrolysis should have a high enough IEC to yield high ionic conductivity with moderate water uptakes to ensure restrained degrees of swelling and acceptable mechanical properties, along with good stability in high pH environments, and low permeabilities to gases, CO_2_ reduction products, and electrolysis intermediates.^19^

With stable polymer backbones (generally without heteroatom links, such as ether groups), an AEM’s stability toward nucleophilic OH^–^ anions (especially at elevated temperatures) is dictated by the cationic group and its hydration, where different headgroups will affect the ionic conductivity, hydration levels, and chemical stability.^26,28,30,38^ For example, AEMs functionalized with trimethylammonium groups have yielded promising thermo-chemical stabilities in both water and CO_2_ electrolyzers.^37^ AEMs based on imidazolium-functional groups also have high OH^–^ conductivities and long-term operando performances;^39^ hence, they are becoming the benchmark AEM for CO_2_E, despite stability limitations at temperatures higher than 60 °C. In addition, cycloaliphatic quaternary ammonium (QA) chemistries (e.g., *N*-methylpiperidinium) have been identified as alkali-stable when hydrated.^40,41^ In a recent study, Ponce-González et al. showed that such chemistries could be introduced into RG-AEMs, yielding promising fuel cell performances.^42^

**Figure 1:**
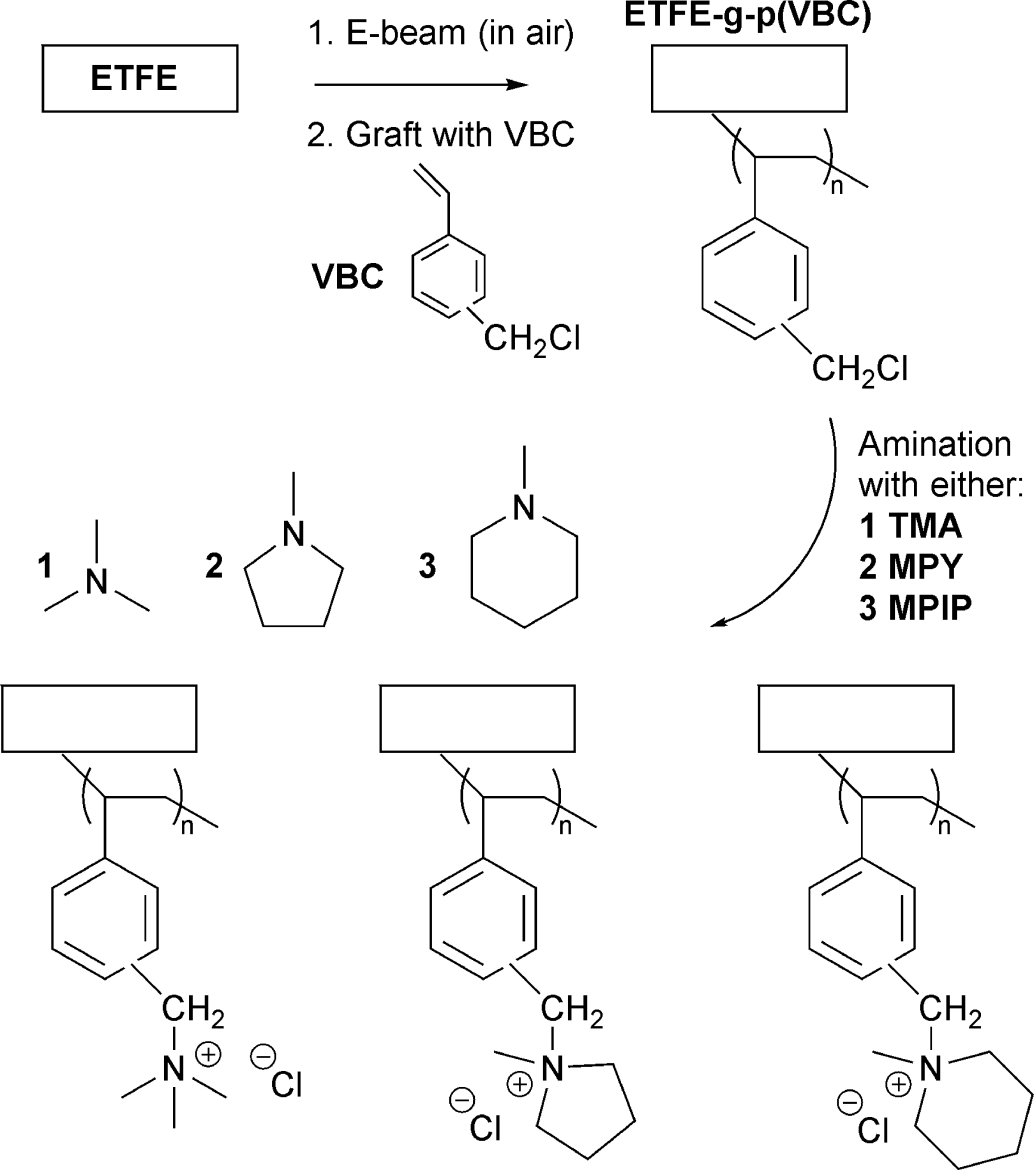
Synthesis of the RG-AEMs with TMA-, MPY-, and MPIP-functionalized groups in the Cl^–^ form. The Cl^–^ anions can be easily exchanged for any other anion desired (HCO_3_^2–^/CO_3_^2–^) via multiple (at least 3×) submersions in aqueous solutions (≥1 M).

Herein, we use the radiation-grafting synthetic platform to conduct a screening of AEM headgroup chemistry for potential CO_2_E applications. RG-AEMs were fabricated by co-grafting vinylbenzyl chloride (VBC) monomers onto electron-beam-activated ethylene-co-tetrafluoroethylene (ETFE) polymer films, followed by amination with either trimethylamine, *N*-methylpyrrolidine, or *N*-methylpiperidine, to yield benzyltrimethylammonium (TMA), benzyl-*N*-methylpyrrolidinium (MPY), and benzyl-*N*-methylpiperidinium (MPIP) RG-AEMs, respectively.^26,42^ This study investigates the effectiveness of such RG-AEMs for CO_2_ electrolyzers, using the zero-gap (MEA) approach at current densities >100 mA cm^–2^. A scheme of the synthesis method via is shown in Figure 1, while a detailed description of the synthesis and characterization methods is found in the Supporting Information (SI).

## Results and Discussion

### Electrode Characterization

Commercially porous Ag membranes with a nominal pore size diameter of 1.2 μm and a thickness of 50 μm and sputtered Cu-GDEs (150 nm) were characterized by XPS and SEM. During the survey scan, carbon and oxygen were detected in the Ag membranes, along with minor peaks indicating chlorine, perhaps related to the formation of AgCl during the electrode preparation. In contrast, as expected, Cu-GDEs showed copper, carbon, and oxygen features (Figure S4). In such measurements, no metallic impurities were observed within the limits of XPS sensitivity that would interfere with the CO_2_E. The SEM images of Cu-GDEs exhibited a spherical morphology (Figure S5), with some roughness variations between the fresh and the postreaction samples.

### Raman Characterization

**Figure 2:**
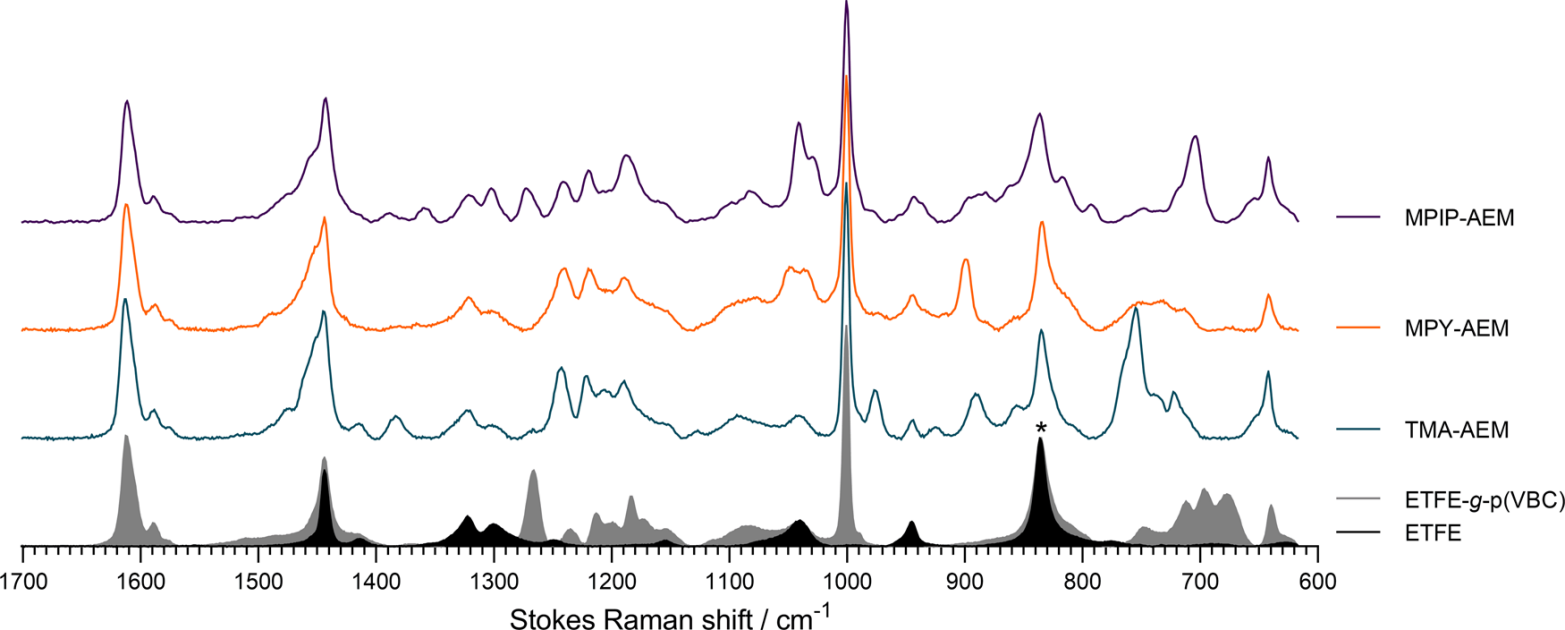
Raman spectra of the 25 μm thick ETFE substrate film (black filled) and the resulting ETFE-g-p(VBC) intermediate grafted membrane (gray filled, average of *n* = 30 spectra taken at random spots across both surfaces), and the final aminated RG-AEMs with line spectra (average of *n* = 10 spectra taken at random spots across both surfaces) stacked higher in the order TMA-AEM (teal), MPY-AEM (orange), and MPIP-AEM (purple). Laser wavelength = 785 nm. * Spectra normalized to the intensity of the ETFE-derived band at 835 cm^–1^ to aid visual comparison. A description of this characterization method can be found in the SI.

The spectra for the pre-aminated ETFE-g-p(VBC) intermediate membrane (degree of grafting 79%) made using the 25 μm ETFE substrate confirms grafting (Figure 2) with characteristic features including bands at 835 and 1444 cm^–1^ (due to the presence of −CF_2_– and −CH_2_– groups in the ETFE substrate, respectively), a grafted poly(VBC)-derived band at 1612 cm^–1^ (aromatic ring quadrant mode), and a band at 1267 cm^–1^ (due to the −CH_2_Cl groups in the grafted poly(VBC) chains).^26^ Raman spectra were collected at 30 random surface sites across both surfaces of the ETFE-g-p(VBC) intermediate membrane (laser spot size ca. 2 μm, while penetrating a few μm into the sample), and the integrated area ratios (areas of the 1612 cm^–1^ bands normalized to the areas of the 835 cm^–1^ bands) were calculated to gauge grafting homogeneity. The band area ratio was recorded as 1.30 ± 0.13, which yields a relative standard deviation = 10%, a quasi-measure of a small but acceptable amount of grafting inhomogeneity (Figure S2). In addition, the Raman spectra of all RG-AEMs confirm successful amination with each amine (Figure 2), based on the disappearance of the poly(VBC)-(−CH_2_Cl)-derived band at 1267 cm^–1^ and the appearance of diagnostic bands for each of the QA groups (756 and 976 cm^–1^ for TMA, 899 cm^–1^ for MPY, and 704 and 1273 cm^–1^ for MPIP).

### Physical Properties of RG-AEMs

The RG-AEMs were characterized for key properties in the Cl^–^ forms to confirm that the ex situ behaviors were as expected. The fundamental physical properties of the ETFE-based RG-AEMs made from 25 μm ETFE films and the three different amines (the equivalent data for the RG-AEMs made from the 50 μm ETFE can be found in Table S3) are summarized. Our results show that incorporating different QA groups onto polymer backbones has a significant effect on the water contents (and swelling) and a lesser effect on the IEC and ionic conductivity (in-plane measurements with the RG-AEM samples submerged in water at 25 °C). MPIP- and MPY-AEMs exhibit higher WU than TMA-AEMs, due to their hydrophilic nature, as reported previously.^42^ In accordance with these results, Koronka et al. reported that AEMs with heterocyclic-QAs would form ionic clusters with more segregated hydrophilic/hydrophobic phases and better channels to transport water, leading to enhanced WU values.^43^ The higher water content variants also tend to swell more (Table 1). For CO_2_E applications, higher IECs and WU AEMs generally lead to higher in situ performances.^44^

**Table 1:** Key Properties for the Synthetized RG-AEMs Made from 25 μm ETFE Films at Room Temperature (Cl^–^ Form)^a^^,^^b^

head group	IEC (mmol g^–1^)	σ_Cl_^–^ (mS cm^–1^)	*T*_hyd_ (μm)	WU (%)	TPS (%)	λ	area swelling (%)
TMA-AEM	2.20 ± 0.02	18 ± 1	56 ± 2	33 ± 1	8 ± 4	8 ± 1	19 ± 3
MPY-AEM	2.07 ± 0.05	23 ± 2	72 ± 2	82 ± 13	46 ± 5	22 ± 4	32 ± 10
MPIP-AEM	2.09 ± 0.07	18 ± 1	69 ± 3	85 ± 19	41 ± 14	23 ± 5	47 ± 18

a The in-plane ionic conductivities (σ_Cl_^–^) in water were measured at 25 °C

b Errors in λ values were calculated from errors in IEC and WU using standard error propagation rules.

**Figure 3:**
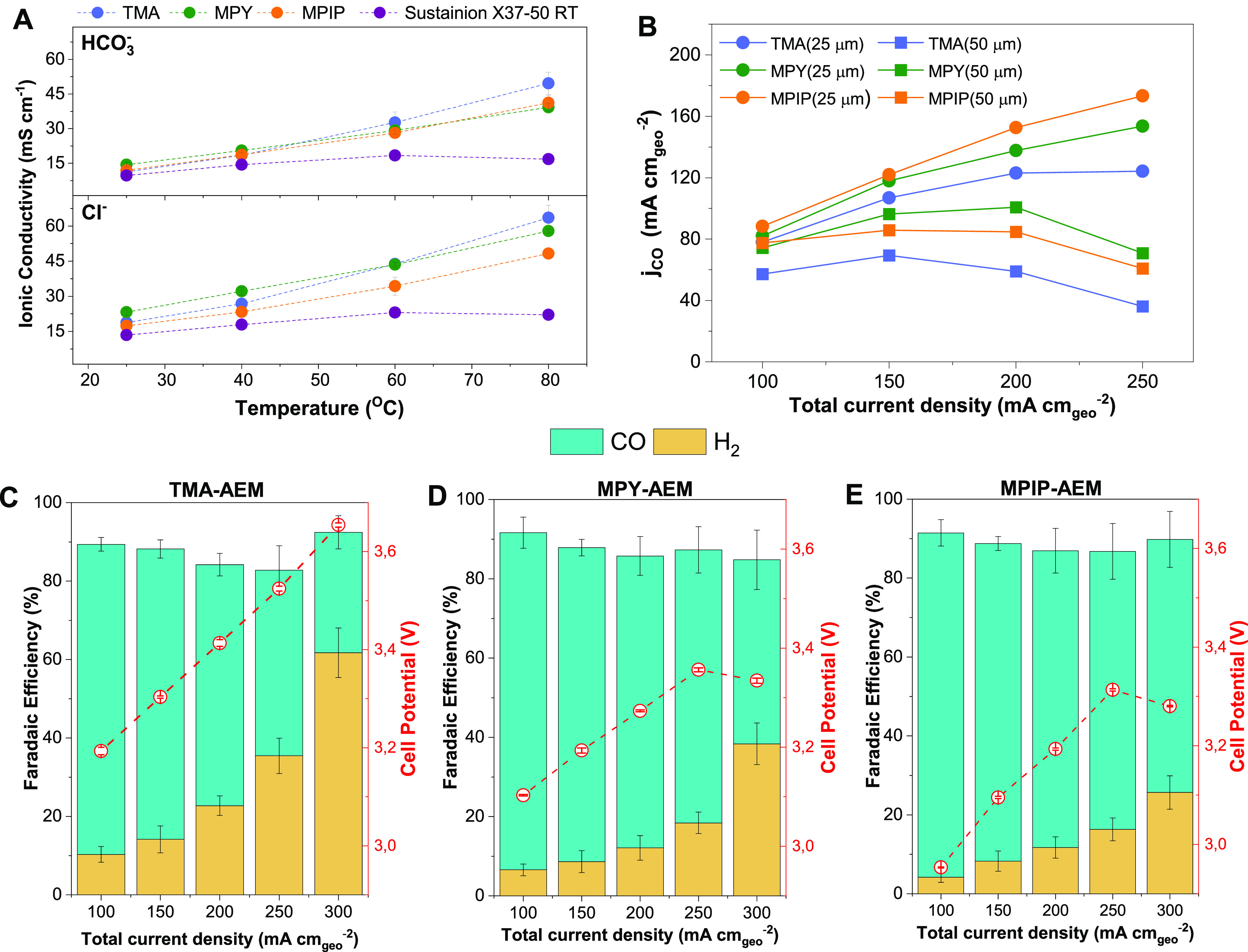
(A) In-plane Cl^–^ and HCO_3_^–^ conductivities (in water) for MPIP-, TMA-, and MPY-AEMs (made from 25 μm ETFE) and Sustainion X37-50 RT at different temperatures. (B) Effect of thickness on the CO partial current density for the RG-AEMs made from 25 and 50 μm ETFE substrate films (as indicated in the legend). (C–E) Product distribution and cell potentials at room temperature for CO_2_E with the different RG-AEMs (made from 25 μm ETFE) as a function of the total current density using Ag electrocatalysts (0.1 M KHCO_3_ anolyte): (C) TMA-AEM, (D) MPY-AEM, and (E) MPIP-AEM. The error bars represent the standard error of the mean for three independent cell tests.

For the RG-AEMs made from 25 μm ETFE, a comparison of the measured Cl^–^ conductivities above 80 °C showed that the MPIP-AEM (Figure 3A) had slightly lower values than the TMA- and MPY-AEMs. We always initially report that values for Cl^–^ form RG-AEMs before any alkaline reagent exposure that risks trace degradations and changes in the nano/micro-morphology. The conductivities of the RG-AEMs in the predominant HCO_3_^–^ were all comparable and slightly lower compared to the Cl^–^ forms (A), owing to differences in ion mobility and diffusivity between ions with different charge densities and hydrated radius.^42,45^

On the other hand, significant differences were observed between these RG-AEMs and those fabricated from thicker 50 μm ETFE (Table S3). The ETFE-g-p(VBC) intermediate membrane made with 50 μm ETFE had a lower degree of grafting (68% compared to 79% with the version made with 25 μm ETFE). The quantitative Raman analysis (Figure S2) also confirmed the lower DOG (degree of grafting) for the thicker ETFE-g-p(VBC) membrane. The higher IECs for the thinner RG-AEMs lead to higher water contents and Cl^–^ conductivities (at 25 °C) compared to thicker analogues. The lower water contents of the thicker versions led to lower CO_2_E performances (B). Hence, all subsequent testing was performed with the thinner RG-AEMs made from the 25 μm thick ETFE.

### Electrochemical Performance

Figure 3(C–E) also shows the results of CO_2_E over Ag electrocatalysts with different RG-AEMs. The experiments were carried out in galvanostatic mode at different current densities ranging from 100 to 300 mA cm^–2^, with CO and H_2_ observed to be the primary products. The total FE was less than 100% due to unaccounted formate (HCOO^–^) being transported to the anolyte (Table S5 and Figure S6). Electro-generated formate is difficult to quantify because of its negative charge, making it susceptible to cross-over through the AEM by covalently bound positive charges and further oxidized to CO_2_ at the anode. In a detailed study of AEM cross-over using the same setup and Ag-electrocatalyst, Larrazábal et al. reported that the FEs toward HCOO^–^ are ca. 20% at higher current densities, which matches the unaccounted-for product FE in this work.^46^

While it is not believed that AEMs themselves directly affect catalytic activity, they do modify the local environment around the cathode catalysts for zero-gap MEA-type cells, particularly in terms of water management, which affects both pH and the mass transfer rates of the CO_2_ at the catalyst’s surface.^47^ Thus, indirectly, AEMs can substantially affect product selectivity, especially related to CO_2_-reduced product(s) vs H_2_. Both MPY-AEM (Figure 3D) and MPIP-AEM (Figure 3E) showed similar product selectivities, with the MPIP-AEM reaching a maximum CO production rate of 5.6 mmol h^–1^ cm^–2^ at 200 mA cm^–2^ (cell voltage of 3.1 V). With these two RG-AEMs, the selectivity toward CO was favored at total current densities <250 mA cm^–2^ (FE_CO_ = 70–87%), but less so at 300 mA cm^–2^ (FE_CO_ < 60%), where HER starts to dominate due to either the cathode flooding or localized mass transfer issues.^48^ MPY-AEM starts to exhibit noticeably lower FE_CO_ values compared to MPIP-AEM at >200 mA cm^–2^. In comparison, lower WU TMA-AEM (Figure 3C) exhibits smaller FE_CO_ values at all current densities compared to MPY- and MPIP-AEM with FE_CO_ < 50% at >200 mA cm^–2^ (along with higher cell potentials).

Water management, the subject of many previous studies on CO_2_E, can be influenced by the nature of the AEM (e.g., QA chemistry)^20,49−51^ and a given AEM’s water content.^52^ It is essential to highlight that WU and the hydration number (λ) are average bulk properties, which does not provide information regarding the nano/micro-distribution of the water-containing channels inside hydrophilic–hydrophobic phase-segregated AEMs. However, λ or WU values still provide insights into the net water transport within CO_2_E cells. Generally, water crosses the AEM by electro-osmotic drag (cathode to anode) and by diffusion or hydraulic permeation (anode to cathode). For our low WU TMA-AEM (λ = 8), we observed faster cathode flooding at *j*_total_ > 200 mA cm^–2^ compared to the higher WU MPY- and MPIP-AEMs (λ > 22), potentially caused by a rise in the water flux to the cathode or a possible decrease in the electro-osmotic drag coefficient.^53^ Similarly, the thickness of the membrane can also influence overall water transport at high current densities since it can influence the hydraulic permeation flux (*J*_HP_) across the AEM (Figure S10). Our results showed that reducing RG-AEM thicknesses increased selectivity and activity independently of the headgroup (Figure 3B).^20^ Moreover, thicker membranes (50 μm ETFE) appeared to enhance electrode flooding as opposed to thinner membranes (25 μm ETFE), due to increased water accumulation at the cathode. For those membranes, a rise in cell potential to compensate for the higher ohmic resistance may accelerate the loss of the GDE hydrophilicity, altering the J_HP_. An in-depth description of the effect of the AEMs properties on water transport can be found in our recent review paper, which explored the effects of membrane characteristics on CO_2_E performance in MEAs.^54^

A comparison of the cell potentials and resistances was performed to understand what caused the difference in performance between the RG-AEMs. Under similar conditions, the measured cell potential (2.9–3.6 V) and resistance (0.2–0.6 Ω cm^2^) were similar to those in the literature for Ag-cathode MEAs.^46,55,56^ Current interrupt measurements (Figure S7) show that the in situ through-plane TMA-AEM resistance of 600 mΩ cm^2^ is significantly higher than that for MPY-AEM (350 mΩ cm^2^) and MPIP-AEM (220 mΩ cm^2^). This contrasts the ex situ HCO_3_^–^ conductivities (Figure 3A), which were similar for all three RG-AEMs; however, only in-plane ex situ measurements were made as it is challenging to get reliable ex situ through-plane conductivities with such thin and highly conductive RG-AEMs. Additionally, even though the RG-AEMs were ion-exchanged in aqueous HCO_3_^–^ solutions, the anions present will be a mixture of HCO_3_^–^ and CO_3_^2–^ (plus traces of OH^–^) due to the equilibria between these anions. At room temperature, the conductivity values for HCO_3_^–^-exchanged RG-AEMs are equivalent to ASRs of 150–250 mΩ cm^2^, which is lower than the in situ values measured (the latter include contact resistances and ionic resistances in the electrodes). Hence, through-plane in situ resistance measurements can be more insightful since relative through-plane resistance trends can be discerned when comparing identical cell set-ups containing different RG-AEMs.

For MPIP-AEM and MPY-AEM, a counter-intuitive phenomenon is observed when transitioning between 250 and 300 mA cm^–2^. The voltage decreases as the cells are pushed to a higher current density. This has been observed previously due to the starvation of CO_2_, resulting in the inability of carbonate anions to form and therefore a switch to predominate OH^–^ anion formation.^46^ This allows the more highly conductive OH^–^ anions to increase in concentration in the AEM, which decreases the device potential as AEMs are most conductive in their OH^–^ forms. However, this starving of CO_2_ also entails CO_2_ electrolysis selectivity decreases in favor of HER. Moreover, incorporating heterocyclic QA groups could provide a hydrophilic–hydrophobic phase-separated morphology with a more efficient ion transport framework due to the electrostatic repulsion of self-aggregated QA groups within hydrophilic domains, resulting in preferential Grotthus-type OH^–^ transport (weakening the OH^–^ binding to the cationic sites).^57−59^ To validate that the RG-AEMs switched to OH^–^ conduction, the anodic CO_2_/O_2_ discharge ratios were measured where they showed a decrease in the ratio at higher current densities, proving this switch to OH^–^ transfer (Figure S9).

**Figure 4:**
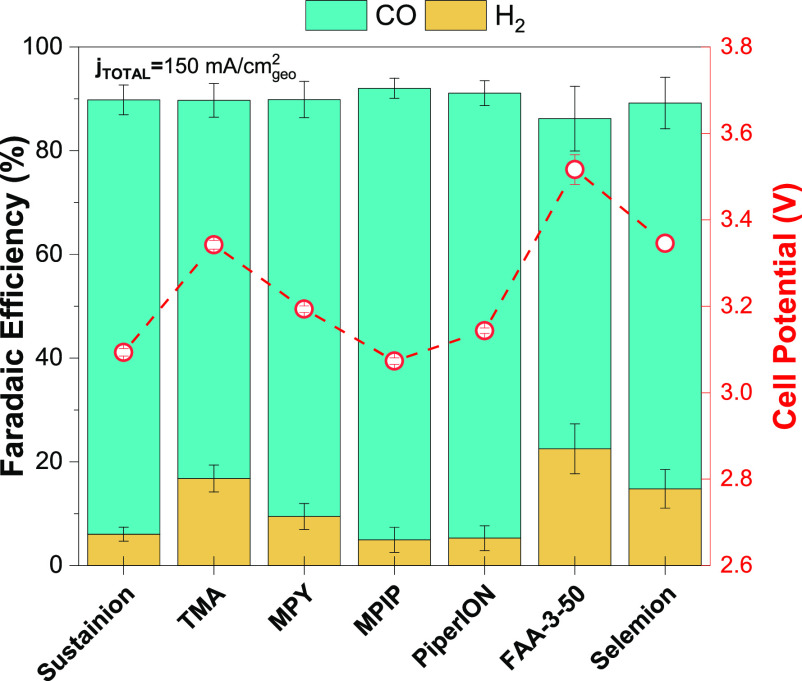
Product distribution and cell potentials for CO_2_E cells operating at *j*_total_ = 150 mA cm^–2^ at room temperature when containing different AEMs (aqueous 0.1 M KHCO_3_ anolyte and Ag electrocatalyst). Sustainion stands for X37-50 RT and Selemion stands for AMV. Error bars represent the standard error of the mean for three independent cell tests for each AEM.

To demonstrate the potential benefits and applicability of this new generation of ETFE-based RG-AEMs for CO_2_E, we conducted comparative studies with other commercial AEMs (Figure 4). Based on our results, MPIP-AEM is competitive with the best commercial membranes, with MPY-AEM being only slightly behind. FAA-3-50, Selemion AMV, and TMA-AEM appear less competitive in terms of cell potential and selectivity under the same experimental conditions at room temperature. The MPIP-AEM reported high FE_CO_ values with low cell potentials, similar to values obtained with Sustainion X37-50 RT and PiperION. In contrast, TMA-AEM and Fumasep FAA-3-50 showed higher cell potentials (270 and 500 mV higher than those of the MPIP-AEM). The fact that all the AEMs tested had different chemistries, cation transfer numbers, and even thickness makes a more direct performance comparison difficult. However, it can be discerned that the AEMs with lower WUs (Fumasep FAA-3-50, TMA-AEM, and Selemion AMV) showed higher potentials and lower CO selectivities, consistent with arguments made vide supra (on the effect of the hydrophobic nature of the headgroups).^20,35^

Additionally, we examined the effect of the anolyte concentration (0.05, 0.1, and 1.0 M) on product distributions, cell potentials, and resistances. By increasing the anolyte concentration, we observed a decrease in the ohmic resistances and a concomitant decrease in cell potential (Figure S11). Such behavior is expected since a higher electrolyte concentration enhances ionic conductivity in the cell. The magnitude of this effect did not appear to be AEM-dependent; thus, the trends discussed in this paper are expected to hold with other anolyte concentrations.

**Figure 5:**
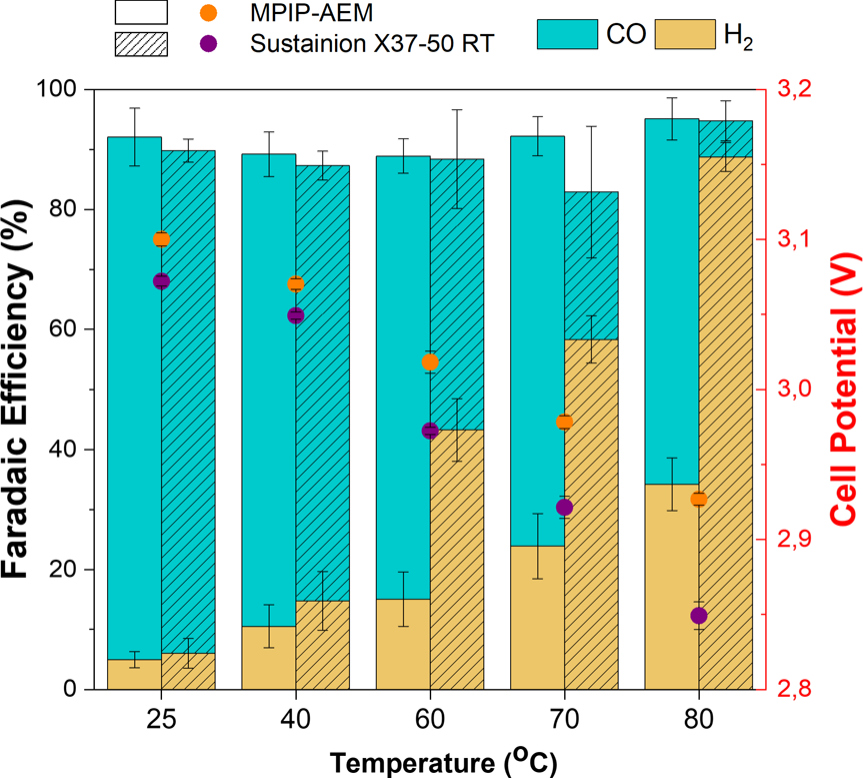
Effect of the operating temperature on the cell potential and Faradaic efficiency for both AEMs E_25_-MPIP and Sustainion X37-50 RT. An Ag electrocatalyst was used as the cathode, and the current density was held at 150 mA cm^–2^ for this experiment. Error bars represent the standard error of the mean for three independent measurements.

The resistive heat produced from any potential commercial CO_2_ electrolyzer entails the necessity of operating at temperatures notably above ambient conditions (>50 °C);^60^ therefore, it is essential to investigate the temperature effects in CO_2_E. Also, elevated temperatures improve CO_2_ reduction kinetics and the ionic conductivity of AEMs (Figure 3A), improving energy efficiencies.^61^ However, excessive temperatures may compromise AEM stabilities due to alkali-derived degradation of the QA groups (or polymer backbones). Hence, we performed experiments at increasing temperatures to compare the thermal stability of MPIP-AEM against the benchmark Sustainion X37-50 RT (Figure 5).

At lower temperatures (<40 °C), the MPIP-AEM resulted in slightly higher operating potentials and similar H_2_ selectivity compared to Sustainion X37-50 RT. However, at 60 °C, the MPIP followed a consistent trajectory in terms of CO_2_-reduced product(s) vs HER as previously discussed. In contrast, Sustainion yielded disproportionate increases in H_2_ selectivity. The cell voltage with Sustainion also commenced more rapid decreases at this temperature. While a decrease in device potential is generally good, this decrease may indicate that the AEM has stability issues (degradation of functional groups and/or mechanical modifications), leading to flooding. Figure 3A also hints the Sustainion AEM degradation above 60 °C as the conductivity starts to decrease above this temperature.

To further investigate the water management effects using the RG-AEMs (MPIP-, MPY-, and TMA-AEM), 24 h tests were employed at 25 °C. All RG-AEMs gave a stable performance with high FE_CO_ (>80%) and constant cell potential throughout the experiments (Figure S12). In addition, the HER remained at a constant level for all AEMs (FE_HER_ = 11–15% for TMA-AEM compared to 3–6% for MPIP-AEM). To further test MPIP-AEM durability, they were tested at 60 °C over 24 h, with again no signs of degradation (Figure S13).

**Figure 6:**
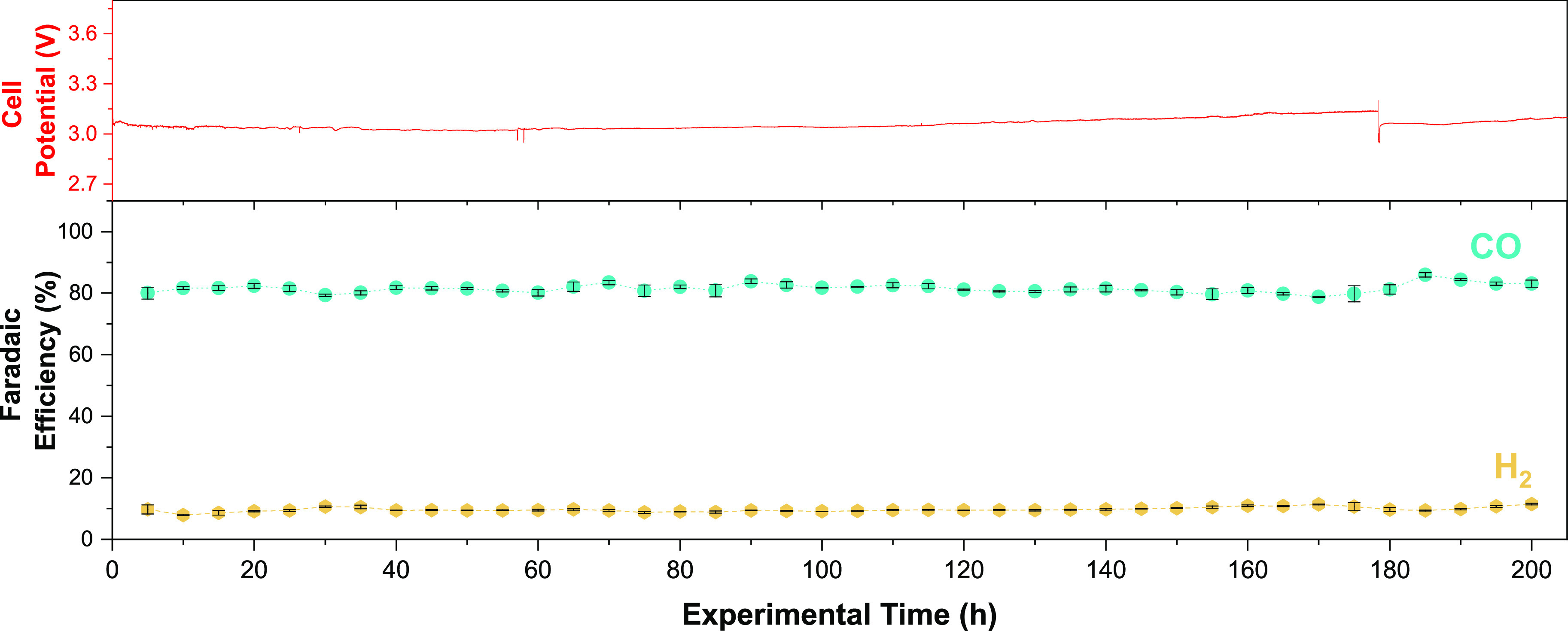
200 h long-term stability test of a room-temperature CO_2_E cell operating at 150 mA cm^–2^ and containing the MPIP-AEM (Ag-based electrocatalyst and 0.1 M KHCO_3_ anolyte). Error bars represent the standard error of the mean for the measurements within 5 h intervals.

It was then decided to test the down-selected MPIP-AEM over 200 h at room temperature (Figure 6). This test showed only minor oscillations in the cell voltage (ca. 100 mV) and stable CO selectivity (FE_CO_ = 80–85%). However, the same experiment with Sustainion X37-50 RT showed less stability, with HER increases occurring over 100 h (Figure S14). While our results do not show the stable operation with Sustainion reported in the literature,^62^ the experiment was replicated multiple times, yielding the same trend. It should be noted that RG-AEMs are substantially easier to handle (more flexible and mechanically robust, without the addition of extrinsic plasticizers) compared to the Sustainion X37-50 RT, which may contribute to the unsuccessful durability tests with this AEMs.

A final feature that was investigated relates to product cross-over across the AEM (when conducting CO_2_ reduction on Cu enabling C_2+_ products). It has been demonstrated that ca. 40% of liquid products can cross the AEMs, caused mainly by diffusion and electromigration.^33^ This experiment aimed to compare the MPIP-AEM developed in this work with different commercial membranes. A switch to the sputtered Cu catalyst led to a CO_2_E cell that produced a range of C_1_ and C_2+_ products, allowing a better understanding of the cross-over properties of the various AEMs.

**Figure 7:**
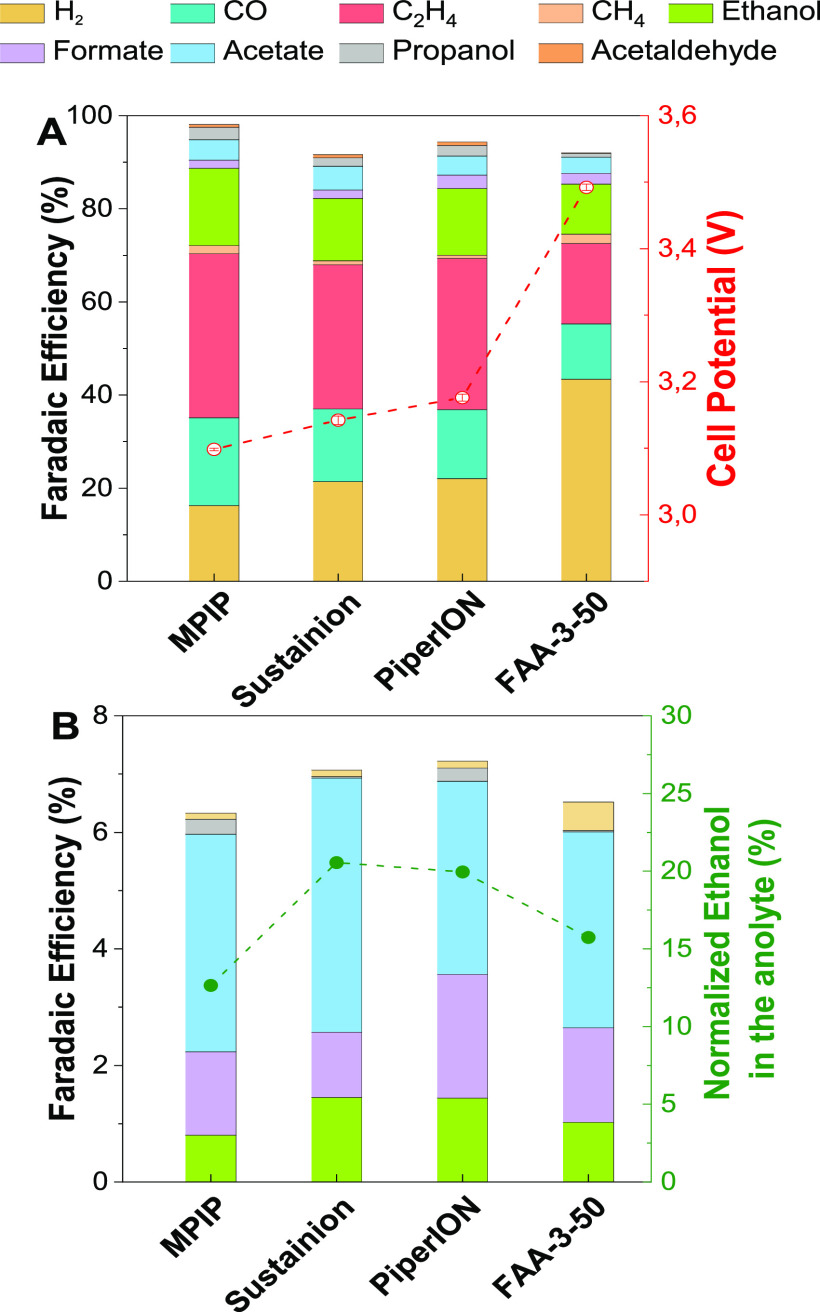
(A) Product distribution and cell potentials obtained with a CO_2_E cell containing a Cu catalyst and various AEMs, when operating at 150 mA cm^–2^ and room temperature. (B) Product distributions detected at the anode. The right-hand axis relates the anodic ethanol Faradaic efficiency as a function of the total ethanol Faradaic efficiency. Sustainion membrane corresponds to X37-50 RT.

Figure 7A shows the results of Cu cathode CO_2_E testing of cells containing various AEMs operating at a constant current density (150 mA cm^–2^). With the similar potentials of all devices, it was reassuring to observe that the general selectivity of all AEMs was consistent. The only notable exception was HER selectivity, which was expected to be primarily related to in situ water management. Regarding H_2_ selectivity, FAA-3-50 gave the highest amount, with MPIP-AEM giving the lowest. In contrast, Figure 7B shows that the liquid cross-over is similar across all the AEMs tested. Since we are using a zero-gap MEA cell, any volatile liquid product (e.g., ethanol, allyl alcohol, and propanol) can evaporate from the GDE (and will be found mainly in the liquid trap with only traces making their way to the anolyte). We observed that MPIP-AEM had the lowest ethanol cross-over compared to the commercial membranes, demonstrating some potential benefits of the RG-AEM morphology and chemistry in regulating neutral product cross-over. Meanwhile, differences in (negatively charged) acetate and formate cross-over were not that noticeable between the different AEMs.

## Conclusions

In this study, RG-AEMs were fabricated from ETFE substrate films of different thicknesses with TMA, MPY, and MPIP quaternary ammonium groups for CO_2_ electrolysis under commercially relevant conditions. These RG-AEMs exhibited desired transport, chemical, and mechanical properties, including high IEC (above 2 mmol·g^–1^), ionic conductivities, moderate water uptake, and low ohmic resistances (<0.6 Ω·cm^2^). The tailored properties of these membranes make them suitable for CO_2_E in MEA configurations, as their mechanical flexibility and robustness make them easily handled during cell assembly while providing long-term stable performance. The operation using thinner membranes with MPIP-head groups yielded the highest CO selectivity (>80%) over Ag-electrocatalysts at higher current densities (with *E*_cell_ = 2.9–3.3 V), owing to improved water and ionic transport within the system. The use of cycloaliphatic headgroups in RG-AEMs proved thermal and chemical stability under different reaction conditions (e.g., operation temperatures and electrolyte), reducing product cross-over and achieving stable operation for at least 200 h for CO_2_E to CO at 150 mA cm^–2^ with high selectivity (80–85%) under RT conditions. Our RG-AEMs exhibit a wide range of properties similar to common commercial AEMs, including the tantalizing possibility of operating at elevated temperatures (80 °C) and demonstrating the potential of a new generation of AEMs for electrochemical CO_2_ reduction applications.

## Supporting Information Available

Synthesis method of RG-AEMs; metrics for RG-AEM characterization; Raman spectroscopy description; preparation and characterization of Cu electrodes; cell configuration; and electrochemical tests; chemical structures of the membranes; information on key properties of common AEMs and our synthesized membranes; XPS and SEM figures of the catalyst; scheme of the reaction setup and electrochemical cell; calculation procedures for FEs and partial current density; HPLC analysis of products; electrochemical tests of 50 μm of RG-AEM and commercial membranes at different current densities; analysis of the CO_2_/O_2_ ratio at the anode; measurements on the effect of electrolyte concentrations; and stability tests (PDF)

## Notes

The raw data collected by the University of Surrey and presented in Figure 2 and Table 1 are available at DOI 10.6084/m9.figshare.21802473 (CC-BY)
