# Supplementary material for: Influence of Headgroups in Ethylene-Tetrafluoroethylene-Based Radiation-Grafted Anion Exchange Membranes for CO2 Electrolysis
Source: ACS Sustain Chem Eng. 2023 Jan 18;11(4):1508–17. doi: 10.1021/acssuschemeng.2c06205 (PMC9890565; doi:10.1021/acssuschemeng.2c06205)
Supplement: Supplementary file 1 — sc2c06205_si_001.pdf [file sc2c06205_si_001.pdf]

- Supporting Information -

Influence of headgroups in ETFE-based radiation-grafted anion exchange membranes for CO_2 electrolysis

Carlos A. Giron Rodriguez,[a] Bjørt Óladottir Joensen,[a] Asger Barkholt Moss,[a] Gastón O. Larrazábal,[a] Daniel K. Whelligan,[b] Brian Seger,[a,*] John R. Varcoe,[b,*] and Terry R. Willson[b]

[a]*Surface Physics and Catalysis (SURFCAT) Section, Department of Physics, Technical University of Denmark, 2800 Kgs. Lyngby, Denmark*

[b]*Department of Chemistry, University of Surrey, Guildford GU2 7XH, United Kingdom*

**Correspondence to: Brian Seger, Email: brse@fysik.dtu.dk and Johm Varcoe, Email: varcoe@surrey.ac.uk*

SI document = 21 pages

Number of Figures = 14

Number of Tables = 6

Sustainion X37-50 RT

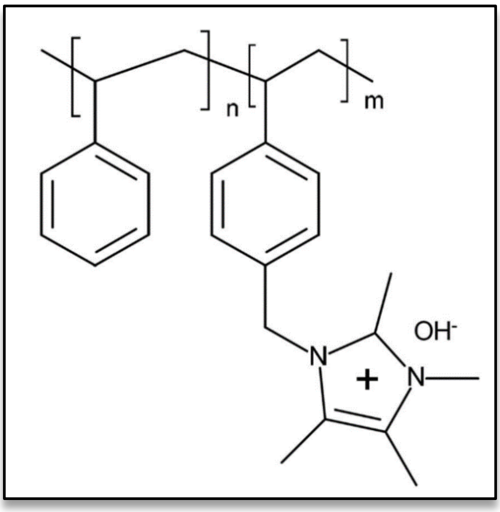

Orion TM1

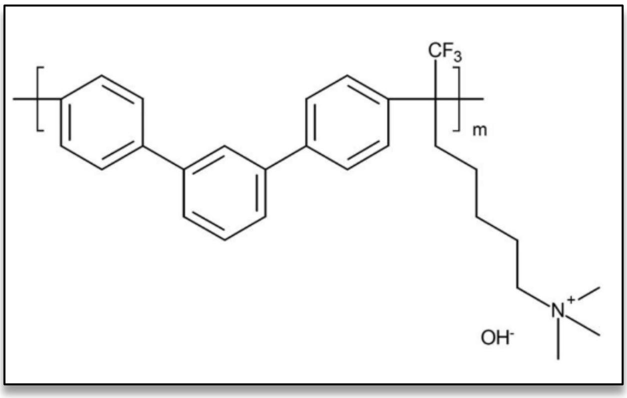

PiperION TP-85

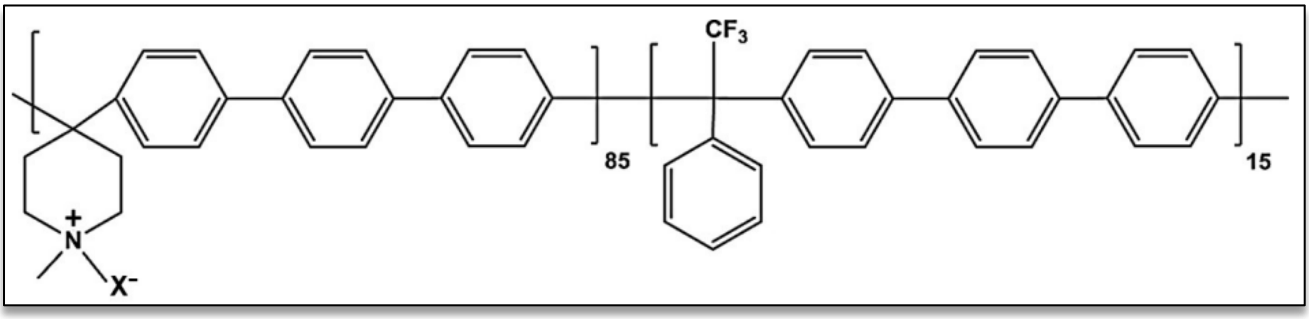

ETFE-TMA

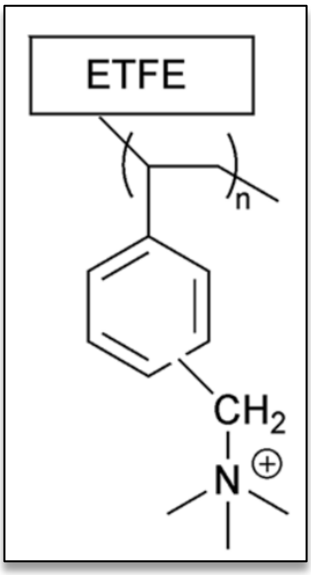

ETFE-MPY

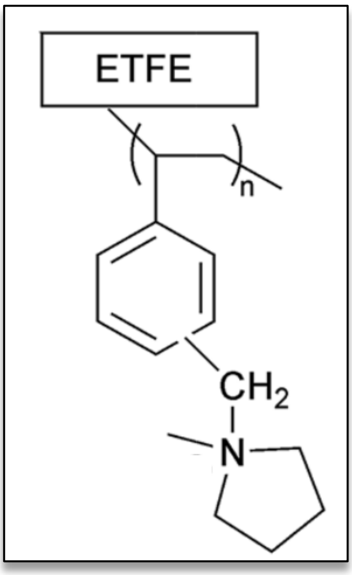

ETFE-MPIP

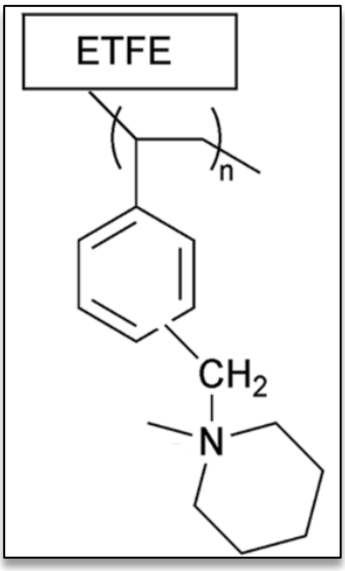

Figure S1 Chemical structures of different AEMs including the ETFE-based radiation-grafted AEMs that were the main subject of the paper.

Table S1 Main key properties of common AEMs for CO_2 electrolysis.

AEM	Counter Ion (anion)	Backbone	IEC (meq g^{-1})	λ	Tensile strength (MPa)	Thickness (μm)	WU (wt. %)	ASR (Ω cm^{-2})	σ (mS cm^{-1})
FAA-3-50[1]	Br^-	N.A	1.6-2.1	N.A	25-40	45-55	10-25	0.6-1.5 (Cl^- form)	3-8 (Cl^- form)
AMVN[2]	Cl^-	PVC	1.85 ± 0.04	N.A	0.3	100-150	15±2	2.5	3.8 (Cl^- form)
X37-50 RT[3]	HCO_3^-	Styrene	2.52	N.A	N.A.	50	>80	0.045 (1 M KOH)	80 (OH^- form)
PiperION	OH^-	Poly(aryl piperidinium)	2.35	N.A	>30	20	50	N.A	150 (OH^- form)
Orion TMI	OH^-	Poly (terphenylene)	2.19	N.A	30	30	33-37	N.A	54 (OH^- form)
m-TPN1[4]	OH^-	Terphenyl	2.1	18	29	15-25	25	0.18	54 (OH^- form)
A201	OH^-	PBI	1.8	25	96 (Cl^- form)	28	44±5	N.A	42
AFI-HNN8-50[5]	OH^-	HMT-PMBI	2.1-2.5	N.A	60 (I^- form)	50	39-50	0.13	>80

*Compiled data correspond to the reported properties from the data sheet of each manufacturer (missing data from the data sheet are found in the references). NA stands for the information not specified/available. Superscript numbers are literature references (see end of SI document).

Materials and chemicals

ETFE polymer films (25 and 50 μm thick) were supplied by Nowofol Kunststoffprodukte GmbH (Germany). VBC (97 % purity, a mixture of 3- and 4-ionomers, 700–1100 ppm nitromethane or 50–100 ppm tert-butylcatechol inhibitors) was purchased from Sigma Aldrich and used as supplied (no removal of inhibitor prior to use). Additionally, 1-octyl-2-pyrrolidone dispersant, *N*-methylpiperidine (99 % purity), aqueous trimethylamine (45 %vol), *N*-methylpyrrolidine (88 %), and acetone were purchased from Sigma-Aldrich (Merck). $AgNO_3$ titration standard analytical solutions (0.02000 ± 0.00006 M) were purchased from Honeywell-Fluka. The ultra-pure water (UPW) used throughout this study was generated using a Purite water purification system (resistivity = 18.2 MΩ cm).

Commercial AEMs Selemion® AMV, Fumasep® FAA-3-50, and PiperION) were purchased from FuelCellStore, while Sustainion® X37-50 RT was purchased from Dioxide Materials. A porous silver membrane with a pore size of 1.2 μm (99.9 % purity) was obtained from Sterterlich Inc. and used as the cathode. The commercial IrO_2-coated carbon paper anode was purchased from Dioxide Materials. $KHCO_3$ (Sigma-Aldrich 99.995 % trace metal basis) and KOH (Sigma-Aldrich 99.95 % trace metal basis) were used as an electrolyte for cell testing or membrane activation solutions.

Methodology for the RG-AEMs synthesis

The RG-AEMs were synthesized via the radiation-grafting peroxidation method. ETFE was selected as a substrate rather than LDPE and HDPE, as quick screening CO_2E cell tests showed that ETFE-based RG-AEMs led to the least parasitic H_2 generation. The VBC was grafted onto electron-beamed ETFE films (40 kGy total dose, 4.5 MeV electron beam) by immersing them in N_2 purged (O_2 free) aqueous dispersion of VBC (5 %vol) in 1-octyl-2-pyrrolidone (1 %vol) and heating at 70 °C for 24 h. After thorough washing in toluene/acetone and drying, batches of RG-membrane [designated ETFE-g-p(VBC)] were obtained with a degree of grafting (dog) = 79 % for the variant made from 25 μm thick ETFE and a dog = 68 % for the variant made

from 50 μm thick ETFE (see Equation S1 in the ESI† for the calculation of dog values). ETFE-g-p(VBC) samples were then aminated separately with either the aqueous trimethylamine (45 %mass, room temperature, 24 h), *N*-methylpyrrolidine (50 %vol, 60 °C, 18 h), or *N*-methylpiperidine (15 %vol, 60°C, 18 h), yielding the desired RG-AEMs.

After the amination, the RG-AEMs were washed multiple times in UPW and soaked in aqueous NaCl (1 M) solutions for 1 h (with at least three solution changes during this period) to ensure the pure Cl^- anion form. After thorough washing in UPW for at least 1 h (with multiple changes in UPW) to remove all excess co- and counter-ions, they were stored in UPW until use. The Cl^- form RG-AEMs can be converted into the predominant HCO_3^- form (with trace amounts of CO_3^{2-} anions) by immersion in aqueous $KHCO_3$ (1 M) for 1 h (multiple changes of solution during this time) followed by thorough washing with UPW (to remove excess co- and counter-ions).

Detailed descriptions for the different steps during RG-AEMs synthesis

Irradiation step: ETFE polymer films (ethylene-tetrafluoroethylene) were supplied by Nowofol Kunststoffprodukte GmbH (Germany) in both 25 and 50 μm thicknesses [designated E25 and E50]. The pre-cut ETFE films were subject to electron-beam irradiation in air to a total absorbed dose of 40kGy using a continuous vertical 4.5 MeV Electron Beam Accelerator, Synergy Health Sterilisation UK Limited, Daventry UK. Post-irradiation the films were immediately stored under solid CO_2 and transported to the University of Surrey where they were transferred to a - 40°C freezer for long-term storage (up to 12 months).

Grafting step: The electron-beamed ETFE films with approximate area of 12 × 12 cm^2 were submerged in an aqueous grafting solution containing 5% vol vinylbenzyl chloride (VBC, 97% purity, mixture of 3- and 4-iomers, 700–1100 ppm nitromethane or 50–100 ppm 4-*tert*-butylcatechol inhibitors, purchased from Sigma Aldrich) and 1 %vol 1-octyl-2-pyrrolidone in deionised water. The grafting solution was then purged with N_2 for 1 h before sealing the vessel and heating it to 70 °C for 24 h. Post-grafting the ETFE-g-poly(vinylbenzyl chloride) grafted films [designated ETFE-g-p(VBC) in the main paper] were thoroughly washed in acetone and toluene to remove any excess VBC as well as any non-grafted poly(VBC) homopolymer that may have formed during the graft reaction. The ETFE-g-p(VBC) membranes were then dried at 50 °C under reduced pressure (vacuum oven) for 3 h to remove all traces of solvent. The degree of grafting (dog, %) of the ETFE-g-p(VBC) intermediate membranes was calculated using the following equation:

$$\mathrm{dog} = \frac{\mathrm{m_g} - \mathrm{m_i}}{\mathrm{m_i}} \times 100\ \% \qquad \textbf{(Eqn. S1)}$$

where m_g is the mass of the ETFE-g-p(VBC) membrane and m_i is the mass of the initial irradiated ETFE film. The dog for the ETFE-g-p(VBC) made from E25 and E50 were 80 % and 68 %, respectively. It was clear that when using the same grafting process (and radiation dose), the thicker ETFE grafted to a lower degree. However, we cannot use higher radiation doses with ETFE as this would lead to RG-AEMs that are too mechanically weak for use in electrochemical cells (especially at elevated temperatures),[6] as the electron-beam treatment process breaks a proportion of the C-C bonds in the ETFE chains (alongside the desired ability to graft monomers).

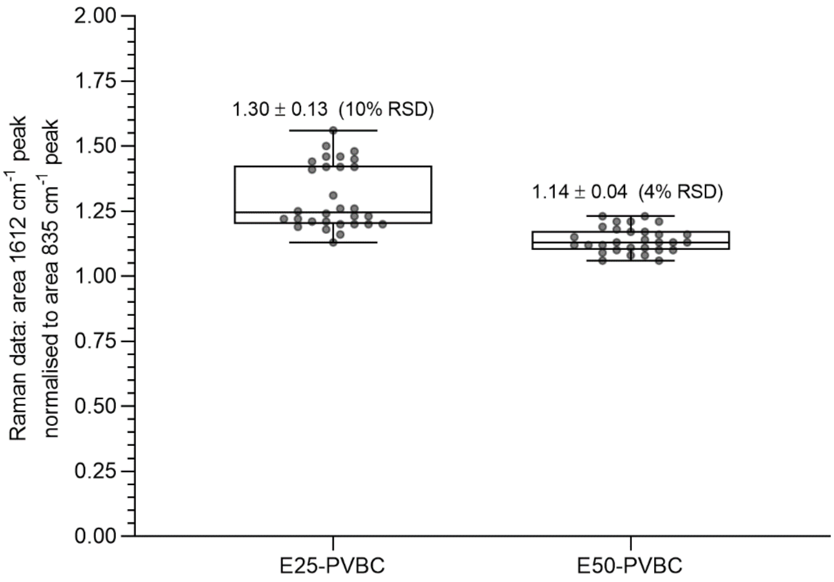

Figure S2 Box plots containing extracted integrated Raman peak area data for spectra recorded on 30 random spots (*ca.* 2 μm diameter laser spot sizes and depths, laser λ = 785 nm) recorded on both sides of the ETFE-*g*-p(VBC) made from both 25 and 50 μm thick ETFE substrate films (E25 and E50). The peak at 1612 cm^{-1} derives from the grafted poly(vinylbenzyl chloride) chains, while the peak at 835 cm^{-1} derives from the ETFE substrate films. Plots show max (top bar), interquartile range (box), median (middle bar), and minimum (bottom bar). The means and sample standard deviations (and relative standard deviations, RSD) are given above the boxes. This data is to evaluate grafting homogeneity (and levels – higher mean peak area ratios for a higher dog). An RSD of 10 % or lower is normal for such lab-fabricated RG-AEMs.

Table S2 The reaction conditions used to aminate the AEMs in this study (of both thicknesses).

	**Amination reaction conditions used**			
**AEM**	**Amine**	**Aqueous concentration (%vol)**	**Temp (°C)**	**Time (h)**
**TMA**	Trimethylamine	45	RT	24
**MPY**	*N*-Methylpyrrolidine	50	60	18
**MPIP**	*N*-Methylpiperidine	15	60	18

Amination step: The ETFE-*g*-p(VBC) intermediate films were subsequently converted to RG-AEMs: TMA-AEM, MPY-AEM and MPIP AEMs *via* submersion in aqueous solutions containing trimethylamine (TMA), *N*-methylpyrrolidine (MPY) and *N*-methylpiperidine (MPIP), respectively. Amination reactions were carried out under various conditions depending on the amine used, with the specific conditions summarised in Table S2. Post-amination all RG-AEMs were thoroughly washed in ultrapure water (UPW) before subsequent heating in fresh UPW at 60 °C for 18 h. This is to ensure that any unreacted amine is removed from the membrane.

Ion-Exchange process to ensure Cl^- forms for storage and initial experiments: To ensure complete conversion to the pristine Cl^- anion form RG-AEMs, the crude, as synthesised, RG-AEMs were submerged in aqueous NaCl solution (1 mol dm^{-3}) for 24 h with the NaCl solution being refreshed three times during this period. The resulting AEMs were then removed and

thoroughly washed with UPW to remove any excess free-ions: both co-ions (Na^+) and excess counter-ions (any Cl^- that are not charge balancing the quaternary ammonium positive charges). The pristine Cl^- anion form RG-AEMs were stored under UPW until use.

RG-AEM characterization

Raman Spectroscopy Raman spectra were recorded on dry samples of the ETFE-g-p(VBC) and final RG-AEMs using a Renishaw InVia Reflex Raman Microscope equipped with a 785 nm IR laser and a 20× (NA = 0.40) objective. All Raman data was collected and baseline corrected using Renishaw WiRE Software (Renishaw PLC, UK), with normalization and integration of band intensities conducted using Spectragryph (Spectroscopy Ninja, Germany).

Ion-exchange capacities (IEC) The ion exchange capacities (IEC) were determined using potentiometric AgCl precipitation titrations. For each RG-AEM in the Cl^- form, a dehydrated sample of known dry mass (m_{dry}) was immersed in 25 mL aqueous $NaNO_3$ solution (1.2 M) and continuously stirred for 16 h. Subsequently, the solution (still containing the RG-AEM sample) was acidified with aqueous 2 mL HNO_3 (2 M) and titrated against aqueous $AgNO_3$ standard solution (0.02000 ± 0.00006 M). A Metrohm 848 Titrino Plus autotitrator equipped with an Ag/AgCl Titrode was used for the dynamic equivalence point titrations (DET). The endpoint was calculated as the peak maxima in the first differential plot of potential *vs.* titrant volume data. IEC was calculated with Equation S2.

$$IEC\ (meq\ g^{-1}) = \frac{E_p \times C_{st}}{m_{dry}} \qquad \textbf{(Eqn. S2)}$$

where E_p represents the endpoint volume, C_{st} is the $AgNO_3$ standard concentration solution, and m_{dry} is the mass of the dry RG-AEM(Cl^-) sample under analysis. This procedure was undertaken on n = 3 samples of each RG-AEM.

Water uptake (WU) and through-plane swelling (TPS) A RG-AEM(Cl^-) sample was removed from UPW storage and the excess surface water was removed by blotting with a filter paper. The hydrated mass (m_{hy}) and thickness (T_{hyd}) were then recorded immediately. Masses were recorded on a 4 decimal place (0.1 mg) analytical balance, and thicknesses were recorded using an outside digital micrometer (precision of ± 2 µm). The RG-AEM(Cl^-) sample was dried under reduced pressure at 50 °C (vacuum oven) for 18 h before the dehydrated mass (m_{dry}) and thickness (T_{dry}) were recorded. All measurements were conducted on n = 3 samples of each RG-AEM(Cl^-). The gravimetric water uptake, through-plane swelling (TPS), and the hydration number (λ) for each sample were calculated using Equations S3 – S5.

$$\mathrm{WU}(\%) = \frac{\mathrm{m_{hyd}} - \mathrm{m_{dry}}}{\mathrm{m_{dry}}} \times 100\ \% \qquad \textbf{(Eqn. S3)}$$

$$\mathrm{TPS}(\%) = \frac{\mathrm{T_{hyd}} - \mathrm{T_{dry}}}{\mathrm{T_{dry}}} \times 100\ \% \qquad \textbf{(Eqn. S4)}$$

$$\lambda = \frac{\mathrm{WU}(\%)/100}{\mathrm{IEC} \times \mathrm{M_{water}}} \qquad \textbf{(Eqn. S5)}$$

where M_{water} corresponds to the molecular mass of water (18.015 g mol^{-1}). Area swelling values were calculated in the same way as TPS values but using the hydrated and dry areas measured at the same time as the thicknesses.

In-plane ion-conductivity The in-plane Cl^- and HCO_3^- anion conductivities of fully hydrated RG-AEM samples between room temperature and 80 °C were measured using a Solartron 1260/1287 combination controlled by ZPlot/ZView software (Scribner Associates, USA). Impedance spectra were collected over a frequency range of 1.0 – 10^6 Hz (10 mV a.c. amplitude) with the samples mounted in a 4-probe BekkTech BT-112 test cell (Alvatek, UK). Test cells containing samples of the Cl^- and HCO_3^- RG-AEM forms were then submerged in UPW. The ionic resistances values, taken as low-frequency *x-(real)*-axis intercepts in the collected Nyquist plots, were used to calculate the conductivities using Equation S6:

$$\sigma\ (\mathrm{S\ cm^{-1}}) = \frac{L}{R\,w\,T} \qquad \textbf{(Eqn. S6)}$$

where *L* corresponds to the working electrode distances (0.425 cm), and *w* and *T* are the width and thickness of the RG-AEM samples, respectively.

Table S3 Summary of the key properties of the E50-based AEMs (Cl^- forms). Errors are sample standard deviations on *n* = 3 repeat measurements (errors in λwater are calculated from the errors in IEC and WU using standard error propagation rules).

	**E50-TMA-AEM**	**E50-MPY-AEM**	**E50-MPIP-AEM**
Ion-exchange capacity IEC (mmol/g)	1.63 ± 0.09	1.56 ± 0.04	1.45 ± 0.04
Fully hydrated thickness (μm)	95 ± 2	106 ± 3	95 ± 2
Conductivity σ in Cl^- at room temperature (mS/cm)	15 ± 2	16 ± 1	12 ± 1
Thickness increase on hydration (%)	20 ± 1	23 ± 2	28 ± 1
Area swelling on hydration (%)	21 ± 1	49 ± 1	39 ± 9
Gravimetric water uptake (% wt.)	18 ± 4	27 ± 2	24 ± 5
λwater (H_2O molecules per exchange site)	6 ± 2	10 ± 1	9 ± 2

Table S4 A summary of key physical and electrochemical properties for Sustainion® X37-50 grade RT in this study.

Ion-exchange capacity IEC (mmol g^{-1})	1.4 ± 0.01
Fully hydrated thickness (μm)	81 ± 2
Conductivity σ in Cl^- at room temperature (mS cm^{-1})	13.5 ± 0.3
Area swelling on hydration (%)	15.1 ± 4.6
Gravimetric water uptake (% wt.)	90.7 ± 14.4
Ion-exchange capacity IEC (mmol g^{-1})	1.4 ± 0.01

Electrode Preparation

Preparation of Cu electrodes for CO_2E to C_{2+} products: Cu-based electrocatalysts were synthesized using physical-vapor deposition (PVD). Layers of 150 nm thick Cu (6N grade) were deposited onto commercial gas diffusion layers (Sigracet 39BB purchased from FuelCellStore) by magnetron sputtering (AJA International) in a vacuum environment (10^{-6} Torr) at a deposition rate of 1 Å s^{-1} under 10 sccm Ar with a sputtering pressure of 2 mTorr. Our Cu-electrodes don't require the addition of any ionomers as binder, so we can evaluate the effects of the membrane's chemistry in the AEM/cathode interface and the reaction, without the potential influence of the binder´s chemistry

Catalyst characterization: Scanning electron microscopy (SEM) of porous Ag and Cu-GDE catalysts was performed using FEI Quanta 200 FEG instrument with an accelerating voltage of 15 kV in secondary electron mode. In addition, X-ray photoelectron spectroscopy (XPS) measurements were carried out in a ThetaProbe instrument (Thermo Fisher Scientific) with monochromatic Al K_α radiation (1486.7 eV) equipped with a hemispherical analyzer. Scans were made in the binding energy range of 0−1400 eV with an analyzer pass energy of 100 eV.

Cell configuration and electrochemical tests: All electrochemical experiments were performed on a commercial electrolysis cell (Dioxide Materials) in a zero-gap MEA configuration. The assembly consisted of loading a fresh AEM (area = 7.4 cm^2) inserted between a cathode (area = 2.25 cm^2) and anode (area = 4 cm^2). PTFE gaskets further sandwiched the MEA device, which helped prevent electrolyte leakage and potential short-circuiting. The system was mechanically pressed, using cell bolts fastened with a torque of 3 N·m to guarantee an uniform and adequate compression. A Bio-Logic VSP 300 potentiostat with a booster channel was used for electrochemical measurements.

The CO_2 (AGA, purity 4.5) flow was set using a mass flow controller (MKS Instruments Inc.) and further humidified by sparging it into a container filled with UPW before being fed to the cathode (standard flow= 40 mL min^{-1}). A liquid trap was installed on the outlet line from the cathodic gas to prevent water from entering the gas chromatograph (GC). This also allowed for the collection of liquid effluent species. The anode was fed with aqueous 0.1 M $KHCO_3$ and continuously recirculated (40 ml min^{-1}) using a diaphragm pump (KNF Neuberger Inc.). The standard conditions for gas flow in this work are 293 K and 1 bar. An Ag/AgCl (3.5 M NaCl internal solution) was employed as the reference electrode. Current interrupt and impedance techniques measured the uncompensated and charge transfer resistances. An illustration of the electrochemical cell and the reaction setup used for these experiments can be found in Figure S3.

The molar outlet flow of the cathodic and anodic streams was measured using a volumetric flow meter (MesaLabs Defender 530) located downstream of the gas chromatograph (GC). The gas product's composition was quantified with a PerkinElmer Clarus 590 GC equipped with a Molecular Sieve 13x, and HayeSep Q packed column using Ar (10 ml min^{-1}) as the carrier gas, and a thermal conductivity detector (TCD). Liquid product analysis was carried out with Agilent Infinity 1260 high-performance liquid chromatography (HPLC), equipped with Aminex HPX-87H column, refractive index (RID), and diode-array (DAD) detectors.

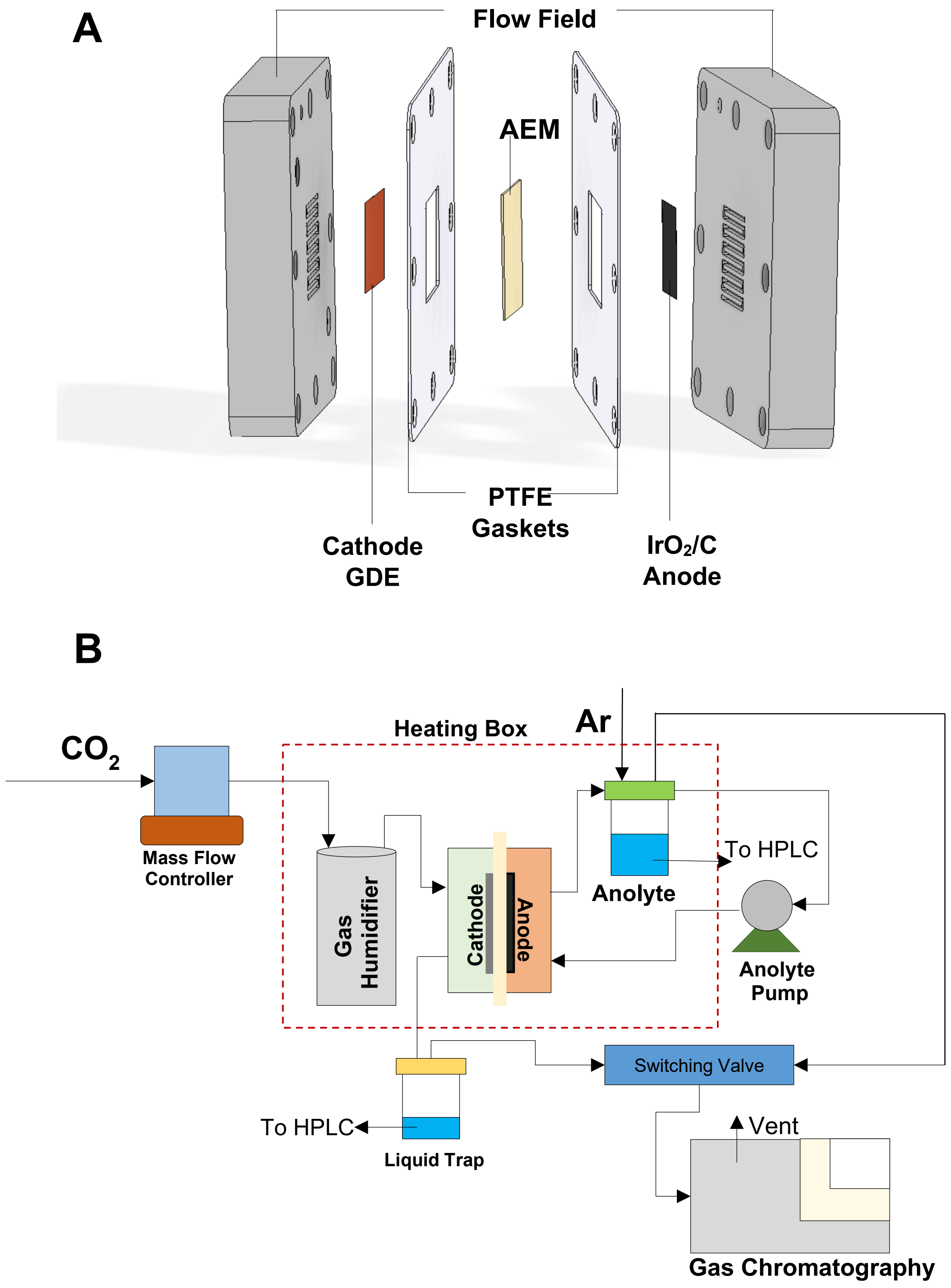

Figure S3 Scheme of **A)** the MEA-type electrolytic cell for electrochemical CO_2 reduction**,** and **B)** Electrolysis set up (use of backpressure regulators or MFM aren't shown in the figure but placed after the electrochemical cell and the gas chromatography).

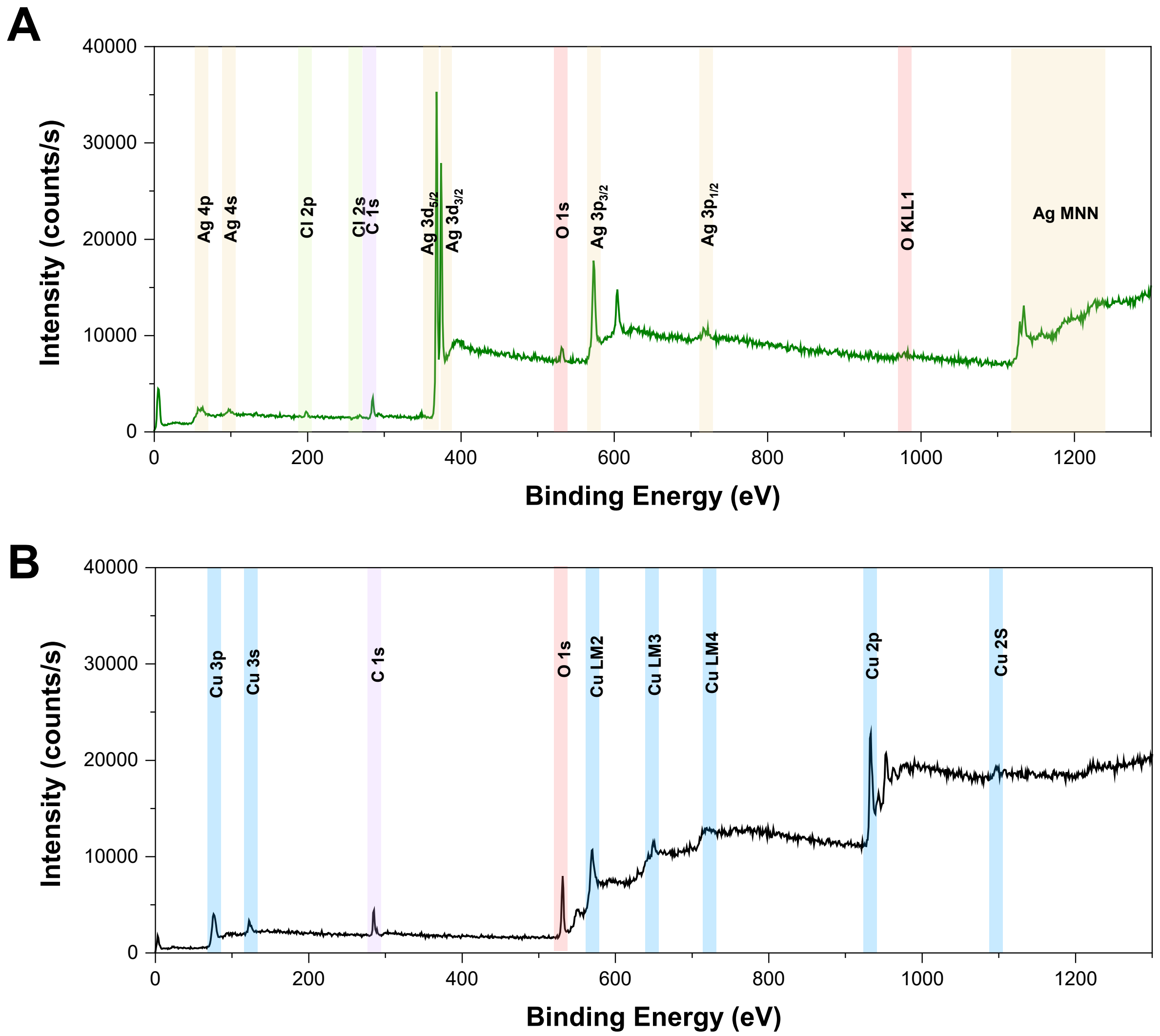

Figure S4 X-ray photoelectron spectroscopy of fresh **A)** Ag-porous based catalyst (Sterlich with a nominal pore size of 1.2 μm) and **B)** Fresh Sputtered Cu (150 nm) deposited in SG39BB. Chlorine and carbon features could be related to deposited traces while preparing the sample.

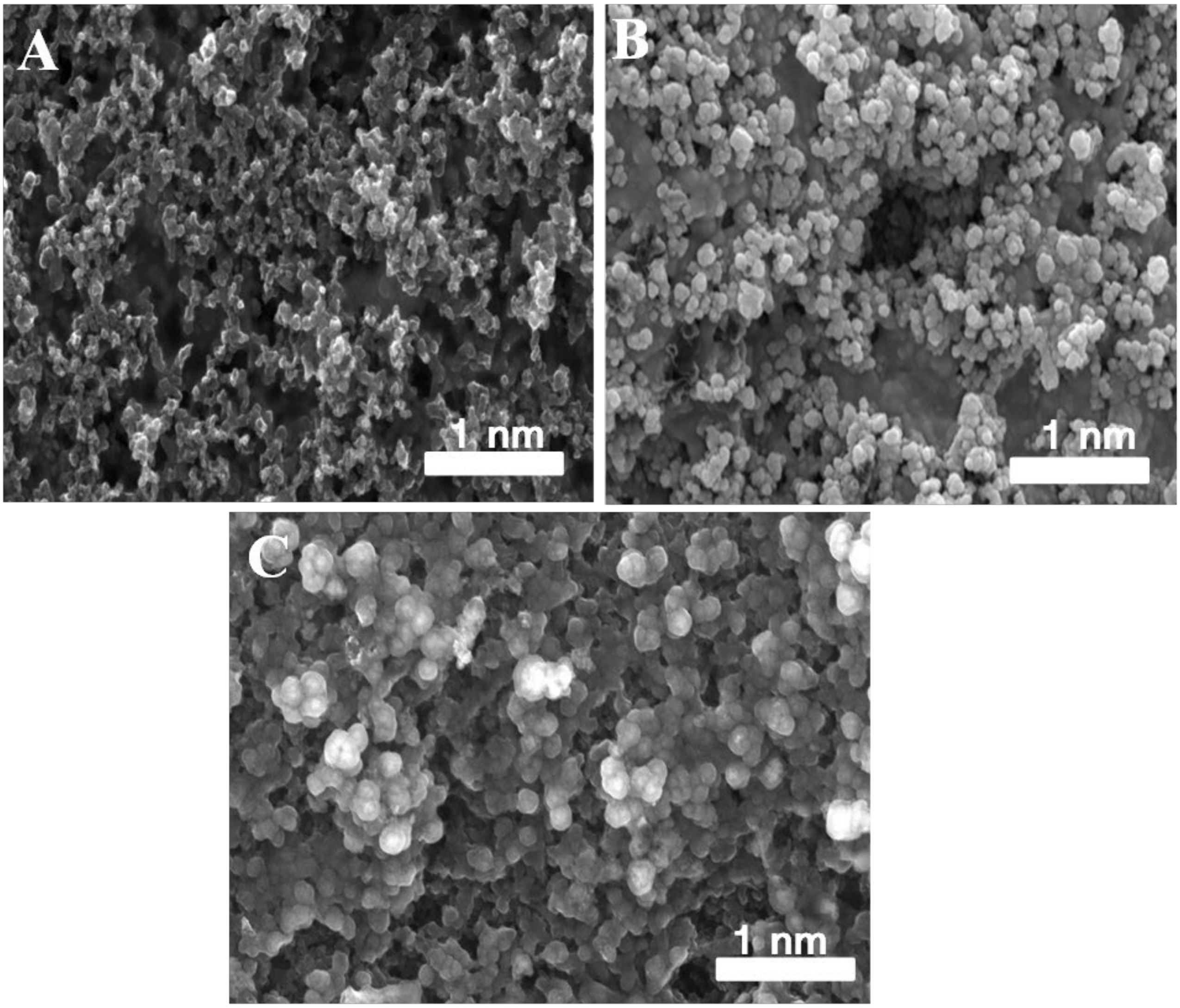

Figure S5 SEM images of the Cu-based catalyst **A)** gas-diffusion layer (GDL), **B)** Cu-GDE coated on microporous carbon layers of the commercial SG-39BB GDL before the electrochemical reaction and **C)** Post-reaction Cu-GDE

Calculation for partial current densities and Faradaic efficiencies

Gas and liquid products are quantified using GC, and HPLC analysis techniques. Partial current density and Faradaic efficiency are two of the methods used in this study to estimate the selectivity and electrochemical performance.

Faradaic efficiency can be defined as the amount of electric charge required to form a desired product over the total charge. It represents the selectivity towards a specific product and can be tuned to improve the conversion and reducing the energy consumption. The calculation of the parameter is expressed in Eqn. S7

$$\mathrm{FE_i} = \frac{\mathrm{z \cdot n \cdot F}}{\mathrm{Q}} * 100\% \qquad (\mathbf{Eqn. S7})$$

where z corresponds to the number of electrons required per mol (z = 2 for CO and H_2), n is moles of the specific products, F is the Faraday´s constant, and Q is the total charge (current × time).

Furthermore, the current density can be defined as the ratio between the total current and the electrode area (geometric or ECSA). The partial current density is associated to the specific product and the product's reaction rate since the electrons transferred in a chemical reaction is proportional to the reaction's extent:

$$\mathrm{j_i} = \frac{\mathrm{I}}{\mathrm{A}} \cdot \mathrm{FE_i} \qquad (\mathbf{Eqn. S8})$$

Calculation of Faradaic efficiency, partial current density and crossover for $HCOO^-$

As mentioned in the main paper, the liquid products analysis was conducted using HPLC for the liquid trap at the cathode and the anolyte. The formate crossover across the membrane and further oxidation over the IrO_2 anode limited the quantification of the total Faradaic efficiency and the partial current density.

Initially, we measured the liquid products at the anolyte and the cathode (collected from the water trap) and correlated the generation of formate in terms of charge. To illustrate these calculations, we take as reference experiments done with the MPIP-AEM (25 μm) at 150 mA cm^{-2} (Figure 3C).

Table S5. Post reaction-liquid analysis and FE-charges for formate quantification

Sample	Volume (ml)	FE_{HCOO^-} (%)	$[HCOO^-]$ (mM)	n_{HCOO^-} (mmol)	q_{HCOO^-} (C)
Anolyte	60	3.64	3.41	0.205	39.5
Cathode	20	0.04	0.0944	1.88×10^{-3}	0.364
				**Total**	39.864

$$\text{Total passed charge (C)} = \mathrm{I} * \mathrm{t} = 1083.24\ \mathrm{C}$$

$$\text{Ratio of corresponded charge (\%)} = \frac{q_{HCOO-}}{q_{total}} = 3.68\%$$

While the crossover and oxidation of the formate is presumed to happen fast (as evidenced in Table S5 and comparison between charge ratios), the quantification of the liquid in post-reaction techniques doesn't provide a good estimation of this product selectivity and reaction extent. Therefore, we assumed that over Ag-based catalysts, the "unaccounted product" is attributed to the CO_2E to $HCOO^-$ and therefore calculated using Eqn.S9 and S10

$$j_{formate} = j_{total} \cdot FE_{formate} \quad \textbf{(Eqn. S9)}$$

$$FE_{formate} = 100\% - FE_{H_2} - FE_{CO} \quad \textbf{(Eqn. S10)}$$

Larrazabal et al.[7] conducted previous experiments to estimate the FE of $HCOO^-$ oxidation over IrO_2/C at 200 mA cm^{-2}, showing that the decrease amount of $HCOO^-$ (in terms of concentration and remained charge) can be related to a FE for $HCOO^-$ ca. 20%.

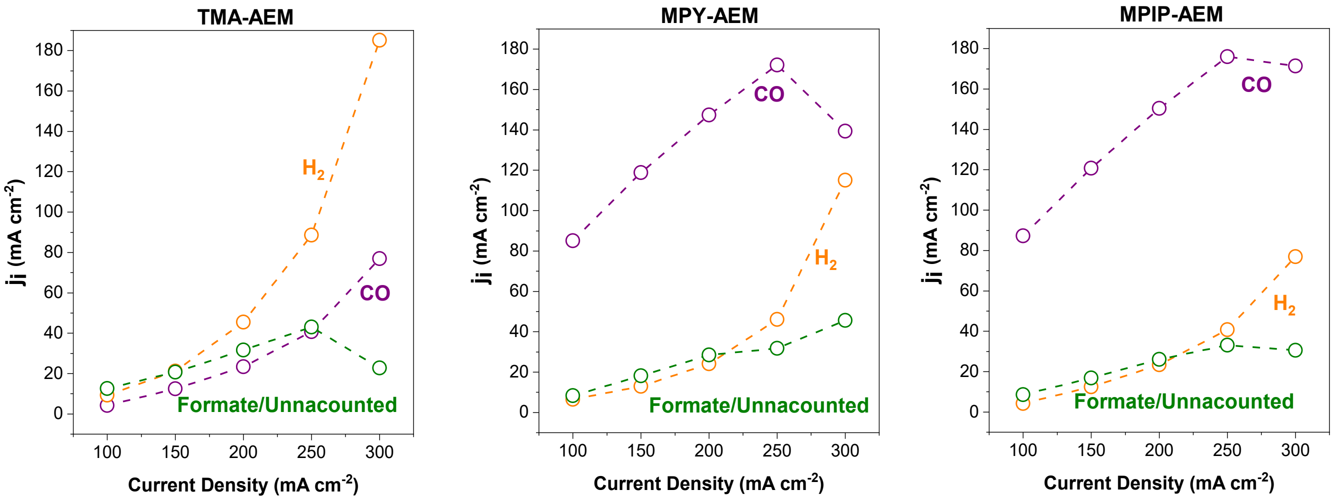

Figure S6 Partial current densities (j_i) for different products as a function of the total current density over Ag-electrocatalyst and 0.1 M $KHCO_3$ anolyte with our different RG-AEMs. Unaccounted products are assumed to be formate. The error bars in such represent the standard error of the mean of three independent measurements.

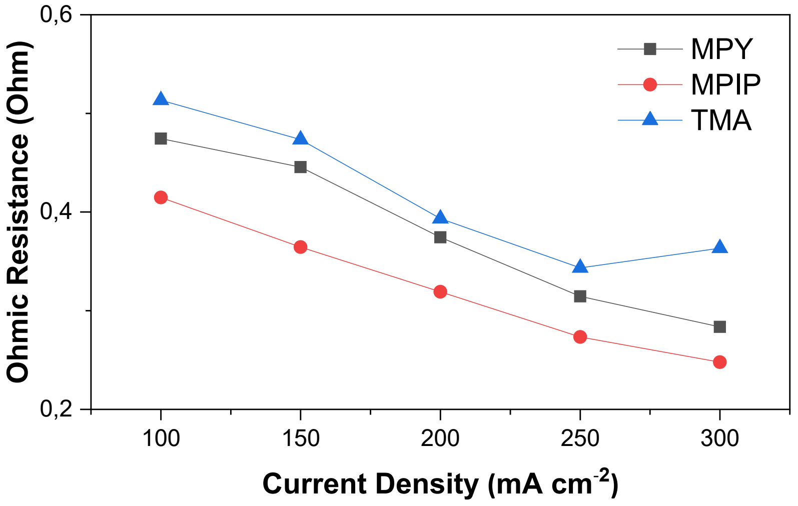

Figure S7 Measurement of ohmic resistances for experiments showed in Figure 3(C-E) for our different RG/AEM's using current interrupt technique (CI).

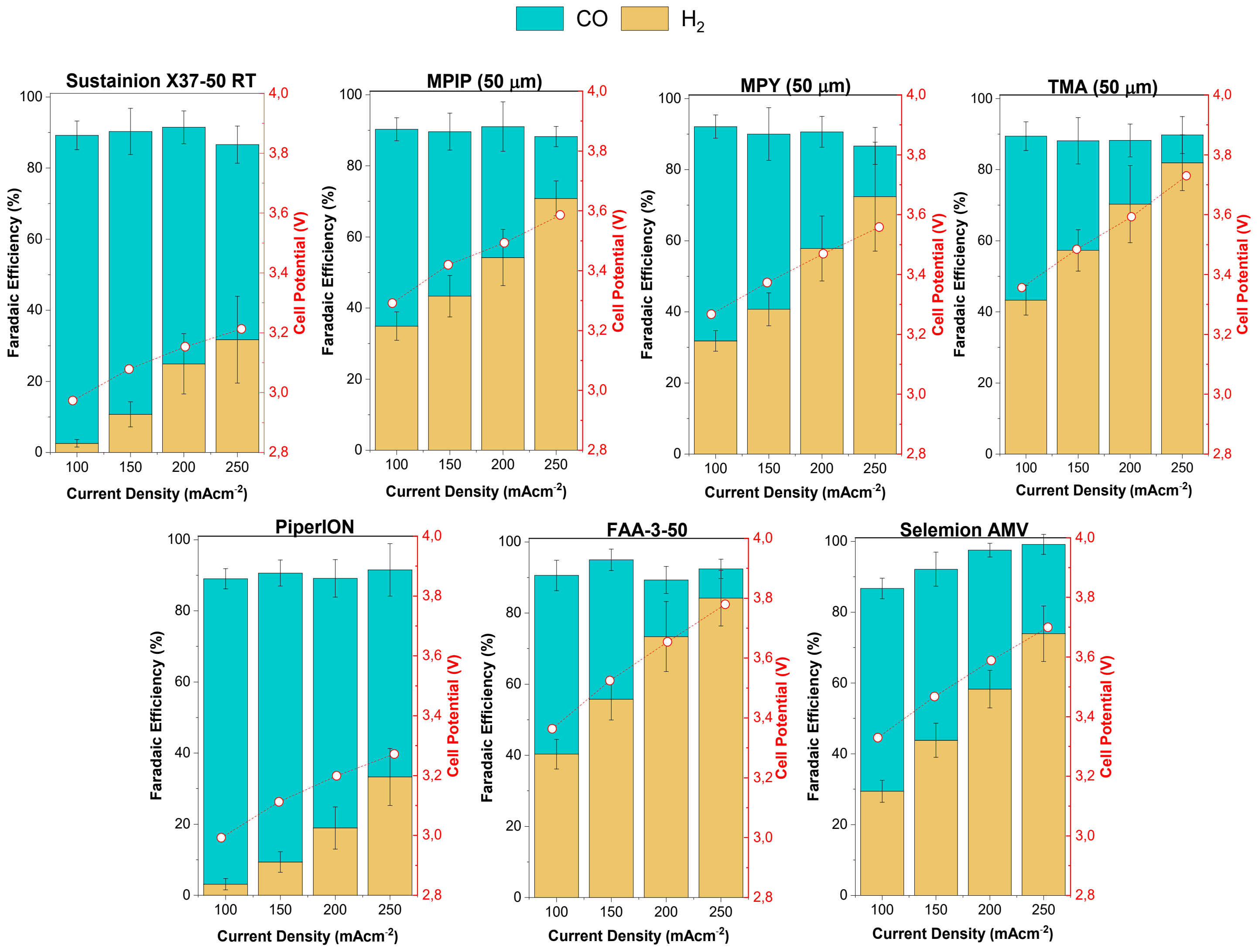

Figure S8 Product distribution and cell potentials at room temperature of different AEMs (including RG-AEM's with thicker 50 µm) as function of the total current density with 0.1 M $KHCO_3$ and Ag-electrocatalyst. The error bars in such represent the standard error of the mean of three independent measurements.

Neutralization reactions and gas evolution at the anode

Neutralization reactions:

$$CO_2 + OH^- \rightarrow HCO_3^-$$

$$HCO_3^- + OH^- \rightarrow CO_3^{2-} + H_2O$$

$$OH^- + H^+ \rightarrow H_2O$$

Gas evolution at the anode:

<u>OER</u>: $2H_2O \rightarrow O_2 + 4H^+ + 4e^-$

Combining the neutralization reaction with the gas evolution at the anode, we have:

$4HCO_3^- \rightarrow 4CO_2 + O_2 + 2H_2O + 4e^-$ **(CO_2/O_2 ratio = 4)**

$2CO_3^{2-} \rightarrow 2CO_2 + O_2 + 4e^-$ **(CO_2/O_2 ratio= 2)**

$4OH^- \rightarrow O_2 + 2H_2O + 4e^-$ **(CO_2/O_2 ratio = 0)**

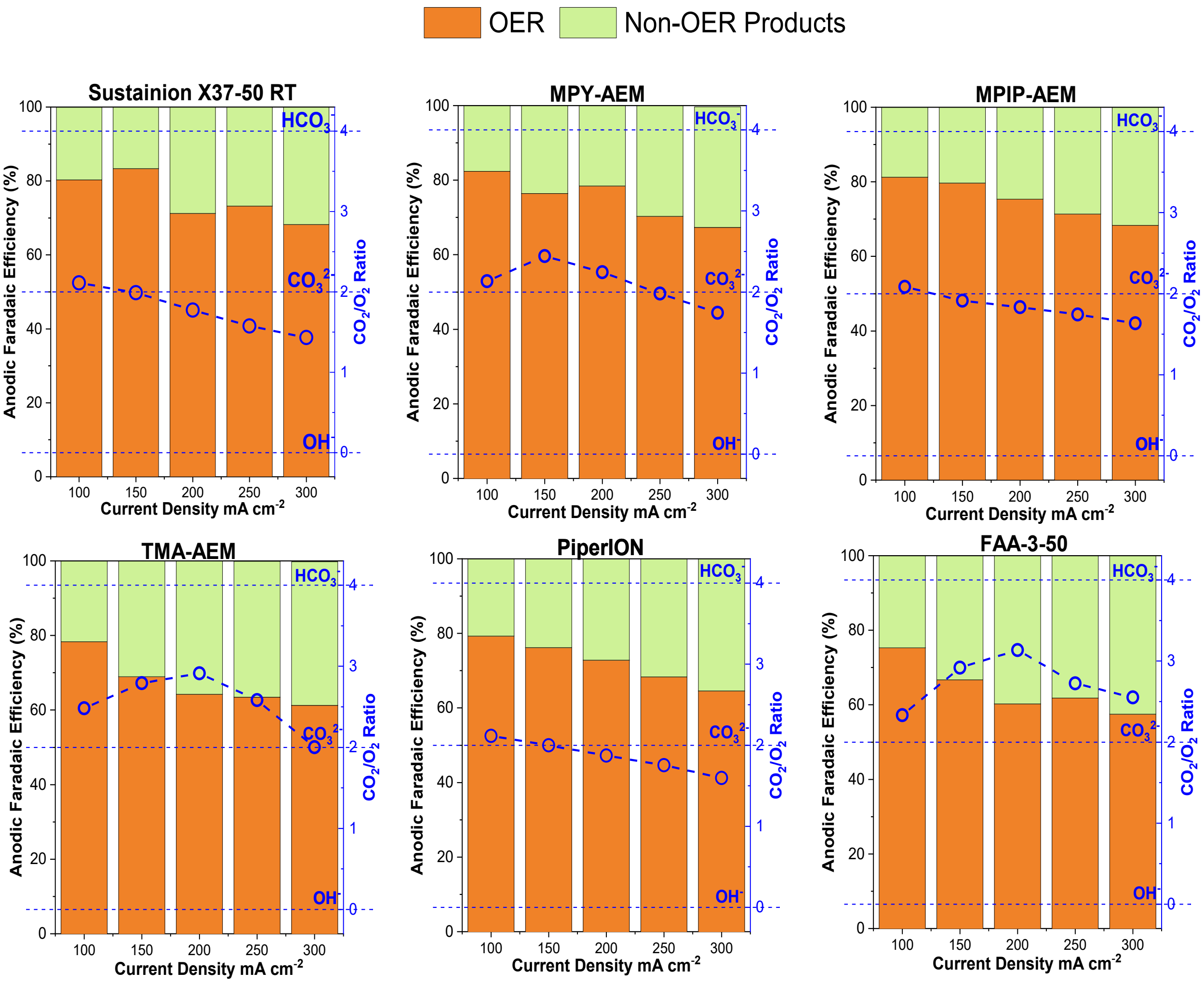

Figure S9 CO_2/O_2 ratio evolved over the anode as a function of total current density for different AEMs performing CO_2 electrolysis with Ag-electrocatalysts and 0.1 M $KHCO_3$

Water Transport across the AEM

Experimental set-up for these ex-situ measurements of the hydraulic water permeation were based on preliminary studies conducted by Duan et. al[8] and Luo et al.[9] for different ion-exchange membranes. By measuring the hydraulic water permeation flux (JHP) in terms of the pressure gradient in the cell, we can correlate the permeability using Equation S11.[10]

$$J_{HP} = \frac{k}{\mu\,\tau} \cdot \Delta P \cdot \frac{\rho_{H2O}}{M_{H2O}} \quad \textbf{(Eq. S11)}$$

where k is the permeability (cm^2), μ the water viscosity (mPa s), ΔP the pressure gradient (bar), τ the membrane thickness (μm).

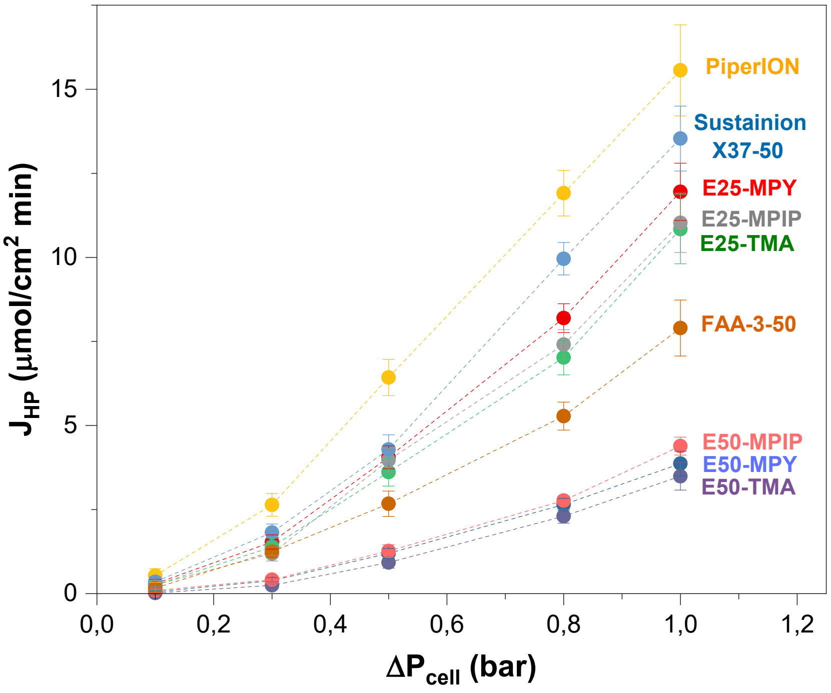

Figure S10 Hydraulic water permeation as function of pressure gradient for different AEMs. (E25 and E50 represents the thickness of the ETFE substrate in μm).

We estimate the permeability by calculating the slope of the curves from Figure S10, and such results are reported on Table S6.

Table S6 Permeability values for hydraulic permeation measurements

AEM	Permeability k (10^{16} cm^2)
MPY-25	2.85
MPY-50	1.38
PiperION	2.10
TMA-25	1.98
TMA-50	1.13
MPIP-25	2.52
MPIP-50	1.37
Sustainion X37-50	2.28
Fumasep FAA-3-50	1.16

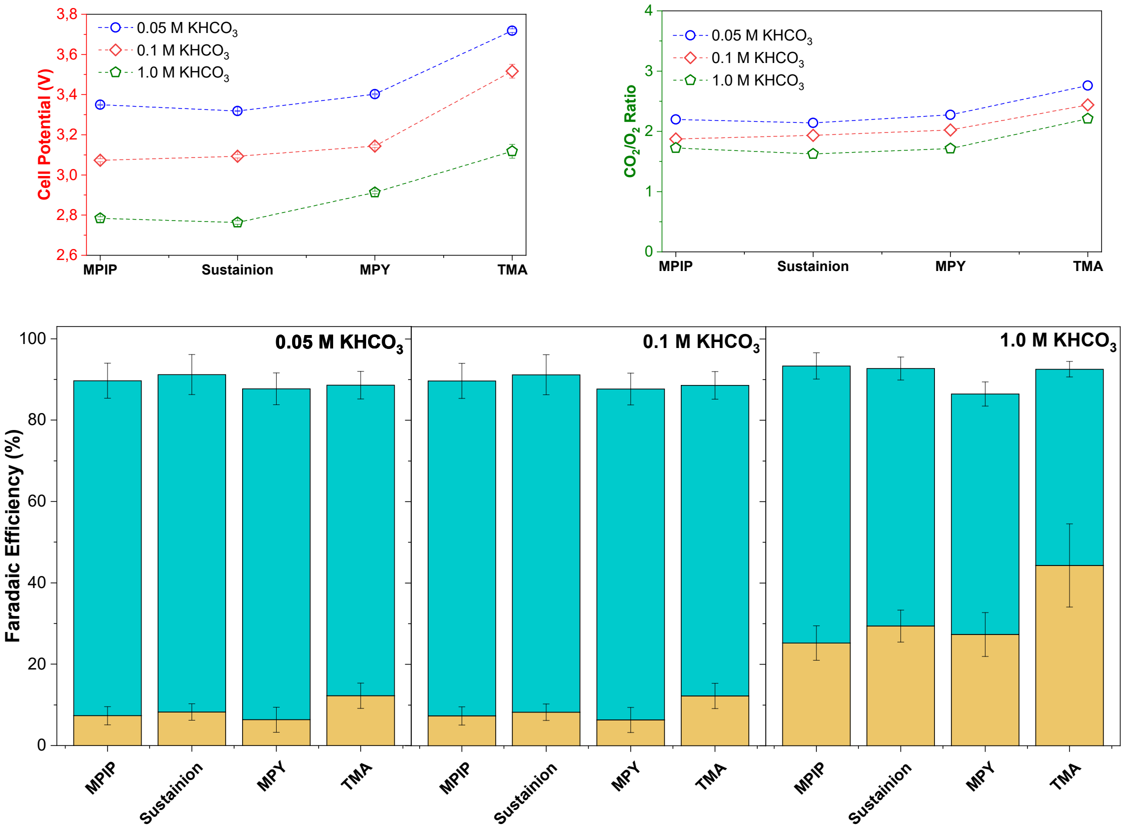

Figure S11 Product distribution, cell potential and CO_2/O_2 ratio using Ag-based electrocatalyst with different RG-AEM´s and commercial Sustainion X37-50 RT, varying the electrolyte concentrations (0.05-1 M $KHCO_3$) at total current density of 150 mA cm^{-2} The error bars in such represent the standard error of the mean of three independent measurements.

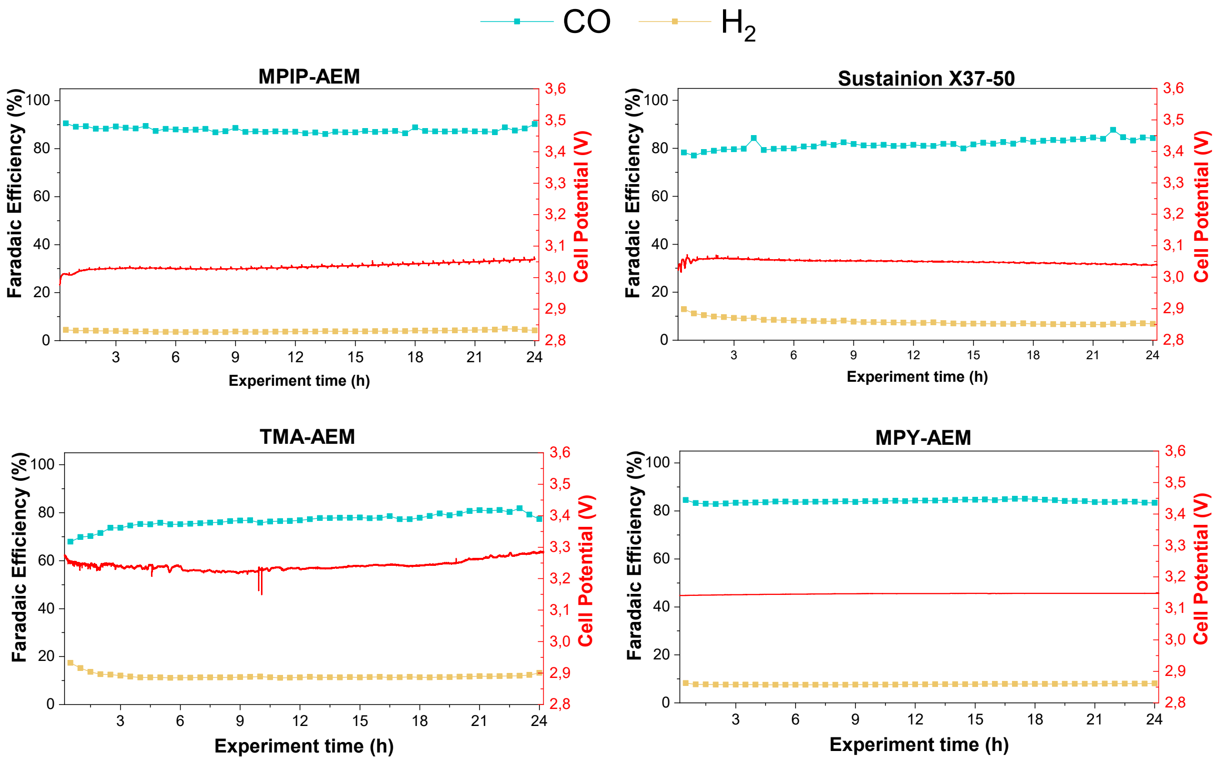

Figure S12 24h Stability-test for different AEMs at room temperature using 0.1 M $KHCO_3$ and Ag-based electrocatalyst. Experimental conditions used are described in the experiment section.

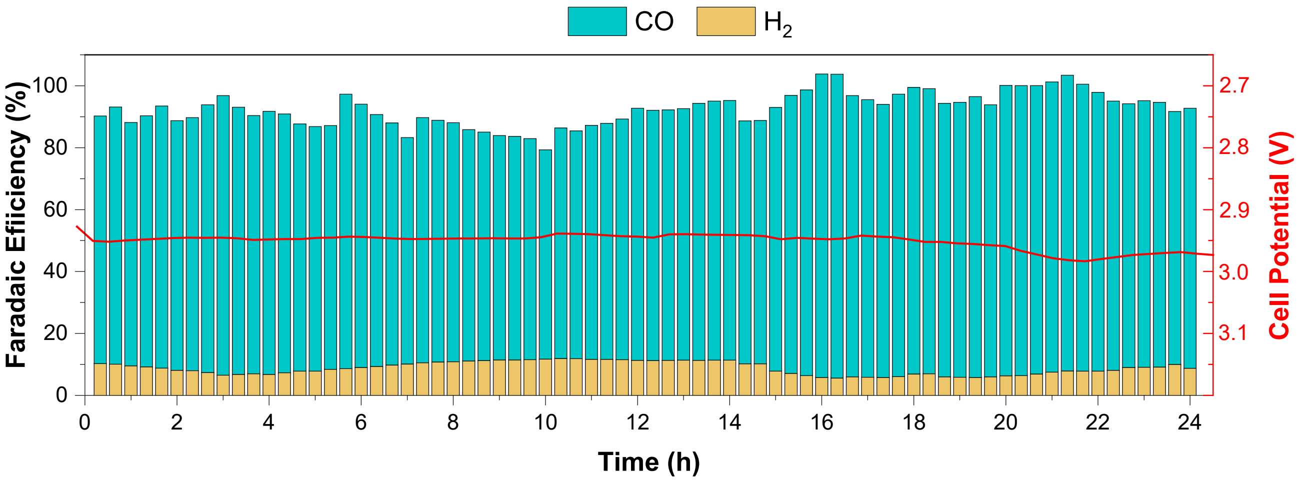

Figure S13 24h Stability-test of MPIP-AEM at 60°C using 0.1 M $KHCO_3$ and Ag-electrocatalyst at 150 mA cm^{-2}

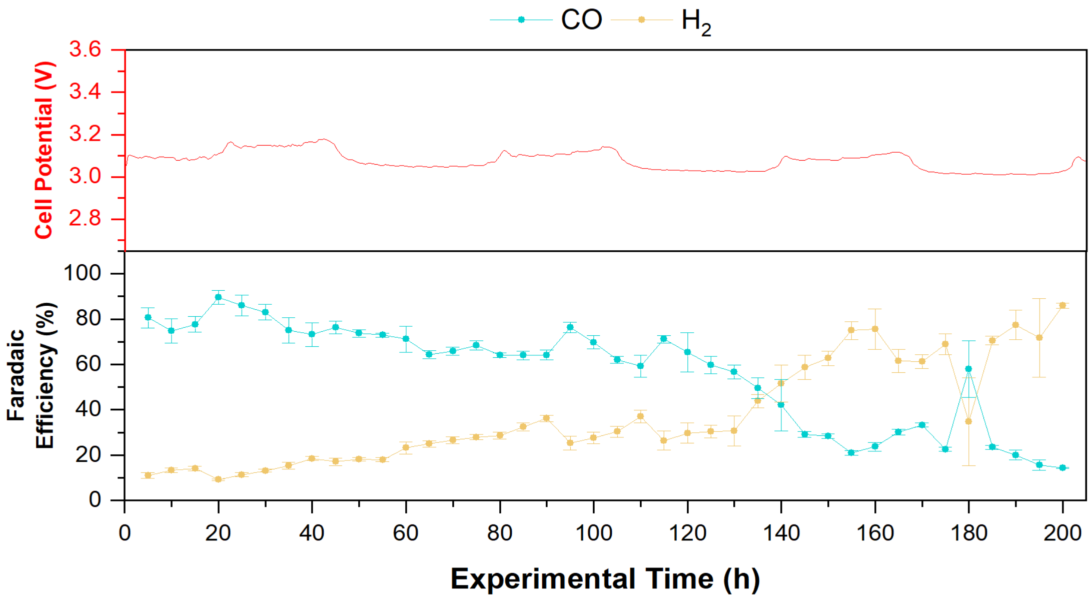

Figure S14 200 h stability test at room temperature using Sustainion X37-50 RT with 0.1 M $KHCO_3$ and Ag-based electrocatalyst.

References

(1) Henkensmeier, D.; Najibah, M.; Harms, C.; Žitka, J.; Hnát, J.; Bouzek, K. Overview: State-of-the Art Commercial Membranes for Anion Exchange Membrane Water Electrolysis. *Journal of Electrochemical Energy Conversion and Storage* **2021**, *18* (2). https://doi.org/10.1115/1.4047963.

(2) Ariono, D.; Khoiruddin; Subagjo; Wenten, I. G. Heterogeneous Structure and Its Effect on Properties and Electrochemical Behavior of Ion-Exchange Membrane. *Mater Res Express* **2017**, *4* (2). https://doi.org/10.1088/2053-1591/aa5cd4.

(3) Kutz, R. B.; Chen, Q.; Yang, H.; Sajjad, S. D.; Liu, Z.; Masel, I. R. Sustainion Imidazolium-Functionalized Polymers for Carbon Dioxide Electrolysis. *Energy Technology* **2017**, *5* (6), 929–936. https://doi.org/10.1002/ente.201600636.

(4) Lee, W. H.; Park, E. J.; Han, J.; Shin, D. W.; Kim, Y. S.; Bae, C. Poly(Terphenylene) Anion Exchange Membranes: The Effect of Backbone Structure on Morphology and Membrane Property. *ACS Macro Lett* **2017**, *6* (5), 566–570. https://doi.org/10.1021/acsmacrolett.7b00148.

(5) Gangrade, A. S.; Cassegrain, S.; Chandra Ghosh, P.; Holdcroft, S. Permselectivity of Ionene-Based, Aemion® Anion Exchange Membranes. *J Memb Sci* **2022**, *641* (August 2021), 119917. https://doi.org/10.1016/j.memsci.2021.119917.

(6) Wang, L.; Brink, J. J.; Liu, Y.; Herring, A. M.; Ponce-González, J.; Whelligan, D. K.; Varcoe, J. R. Non-Fluorinated Pre-Irradiation-Grafted (Peroxidated) LDPE-Based Anion-Exchange Membranes with High Performance and Stability. *Energy Environ Sci* **2017**, *10* (10), 2154–2167. https://doi.org/10.1039/c7ee02053h.

(7) Larrazábal, G. O.; Strøm-Hansen, P.; Heli, J. P.; Zeiter, K.; Therkildsen, K. T.; Chorkendorff, I.; Seger, B. Analysis of Mass Flows and Membrane Cross-over in CO_2 Reduction at High Current Densities in an MEA-Type Electrolyzer. *ACS Appl Mater Interfaces* **2019**, *11* (44), 41281–41288. https://doi.org/10.1021/acsami.9b13081.

(8) Duan, Q.; Wang, H.; Benziger, J. Transport of Liquid Water through Nafion Membranes. *J Memb Sci* **2012**, *392–393*, 88–94. https://doi.org/10.1016/j.memsci.2011.12.004.

(9) Luo, X.; Wright, A.; Weissbach, T.; Holdcroft, S. Water Permeation through Anion Exchange Membranes. *J Power Sources* **2018**, *375*, 442–451. https://doi.org/10.1016/j.jpowsour.2017.05.030.

(10) Garg, S.; Giron Rodriguez, C. A.; Rufford, T. E.; Varcoe, J. R.; Seger, B. How Membrane Characteristics Influence the Performance of CO_2 and CO Electrolysis. *Energy Environ Sc.* ***2022**, 15, 4400-4469*. https://doi.org/10.1039/d2ee01818g.
